# MYB and HIF1α crosstalk drives hypoxia-induced transcriptional reprogramming and adaptive signaling alterations in pancreatic cancer

**DOI:** 10.1016/j.canlet.2025.217916

**Published:** 2025-07-16

**Authors:** Shashi Anand, Kunwar Somesh Vikramdeo, Mohammad Aslam Khan, Seema Singh, Ajay Pratap Singh

**Affiliations:** a Cancer Center and Research Institute, University of Mississippi Medical Center, Jackson, MS, USA; b Department of Cell and Molecular Biology, University of Mississippi Medical Center, Jackson, MS, USA; c Department of Pharmacy and Pharmaceutical Sciences, St. Jude Children’s Research Hospital, 262 Danny Thomas Pl, Memphis, TN, USA

**Keywords:** MYB, HIF1α, Hypoxia, Pancreatic cancer, Transcriptional reprogramming

## Abstract

Pancreatic cancer is an aggressive malignancy, characterized by extensive desmoplasia and a hypoxic tumor microenvironment that contributes to therapy resistance. MYB, a proto-oncogene encoding a transcription factor, plays a crucial role in pancreatic tumor growth and metastasis. Recently, we also revealed a role of MYB in hypoxic survival of pancreatic cancer cells by promoting metabolic reprogramming through interaction with HIF1α, modulating its expression and binding to glycolytic gene promoters. In this study, we investigated how hypoxia influences the genome-wide occupancy of MYB using chromatin immunoprecipitation sequencing (ChIP-seq), and whether this effect is modulated by its interaction with HIF1α. In addition, we examined the genomic distribution of HIF1α in presence and absence of MYB and the impact of their crosstalk on the transcriptional output and associated signaling alterations by RNA sequencing (RNA-seq) and pathway analyses. Our findings show that hypoxia induces significant changes in the genomic distribution of MYB, which is partly dependent on HIF1α. We also demonstrate a significant impact of MYB on HIF1α genomic binding, identifying a subset of hypoxia-induced genes, co-regulated by MYB and HIF1α. These genes are involved in key metabolic and oncogenic signaling pathways critical for hypoxic adaptation. Together, these findings highlight the functional significance of reciprocal crosstalk between MYB and HIF1α, providing novel mechanistic insights into pancreatic cancer pathobiology.

## 1. Introduction

Pancreatic cancer is a devastating disease characterized by its aggressive nature and extremely poor prognosis [1]. A key hallmark of pancreatic cancer is extensive desmoplasia that creates a highly hypoxic tumor microenvironment [2]. Hypoxia also leads to the formation of disorganized blood vessels, further exacerbating the tumor’s inability to receive adequate oxygen and nutrients [3]. Additionally, the hypoxic conditions contribute to the invasive nature of pancreatic cancer cells, facilitating their spread to surrounding tissues [4]. Hypoxic microenvironment also plays a critical role in resistance to chemo- and radiation-therapy since lack of oxygen availability restricts the production of reactive oxygen species (ROS) required for mediating the DNA damage [5,6]. Hypoxia is also shown to impair immune cell function, which further helps in the relentless tumor growth [7]. Therefore, elucidating the molecular targets and signaling pathways that mediate hypoxic adaptation is of paramount importance and holds significant promise for the discovery of novel therapeutic strategies.

Hypoxia-inducible factors (HIFs) play a critical role in the adaptation of cancer cells under hypoxia by inducing transcriptional reprogramming [8,9]. Recently, we identified MYB as a novel player in the hypoxic survival of pancreatic cancer cells through its interaction with HIF1α [10]. MYB is a proto-oncogene encoding a transcription factor expressed in immature hematopoietic cells across all lineages, where it plays a crucial role in maintaining their undifferentiated proliferative state [11]. Its expression has also been reported in colonic and neural stem cells as well as several hematologic and solid tumors [12–14]. Previously, we demonstrated a role of MYB in pancreatic tumor growth and metastasis [15]. We also showed that MYB promotes desmoplasia by enhancing communication between pancreatic cancer and stellate cells [16]. Further investigations into MYB-regulated gene networks and the secretome of pancreatic cancer cells revealed its broader pathological relevance [17,18]. Our recent study discovered that hypoxia induces MYB expression through a post-transcriptional mechanism, and MYB facilitates hypoxic survival by promoting metabolic reprogramming. We observed that MYB interacts with HIF1α, modulating its expression and binding to glycolytic gene promoters [10]. However, a broader role of MYB in hypoxia-induced transcriptional reprogramming through its crosstalk with HIF1α remained undefined.

In this study, we investigated the impact of hypoxia on the genome-wide chromatin occupancy of MYB and if HIF1α plays a role in defining its genomic specificity. Additionally, we also studied the role of MYB in the genomic distribution of HIF1α. Our findings show that hypoxia induces changes in MYB genomic occupancy and this redistribution is partly dependent on HIF1α. Furthermore, we show that MYB and HIF1α share a significant number of gene targets and MYB plays a critical role in selective binding of HIF1α in the promoter regions of several of these shared gene targets. MYB and HIF1α co-regulated genes support activation of several important metabolic and oncogenic signaling pathways. We also show the clinical significance of select top co-regulated genes in pancreatic tumor aggressiveness and poor patient survival. Collectively, our data unfolds a novel reciprocal regulatory crosstalk between MYB and HIF1α, driving optimal adaptation of pancreatic cancer cells under hypoxia.

## 2. Methods

### 2.1. Cell culture and treatment conditions

Pancreatic cancer (PC) cells MiaPaCa, and BxPC3 were cultured in RPMI medium (Cytiva, Marlborough, MA, USA) and Panc-1 cells were grown in DMEM medium (Life Technologies, Carlsbad, CA, USA). All media were supplemented with 10 % (v/v) FBS (Atlanta Biologicals, Lawrenceville, GA, USA), 1 % penicillin, and streptomycin (Life Technologies, Carlsbad, CA, USA). The cultured cell lines were routinely tested for Mycoplasma and treated with anti-mycoplasma reagent intermittently. For hypoxia treatment, cells were seeded in either 60 mm or 150 mm culture dishes, allowed to grow for 24 h, and then subjected to 1.0 % O_2_ in a hypoxia chamber equipped with an oxygen controller (BioSpherix, Parish, NY) for respective time intervals.

### 2.2. Generation of CRISPR/Cas9 knockout cell lines

MYB knockout (MiaPaCa and Panc1) and HIF1α knockout (MiaPaCa, Panc1, and BxPC3-MYB) pancreatic cancer cells were generated using the Alt-R CRISPR-Cas9 gene-editing system (Integrated DNA Technologies, Coralville, Iowa, USA) as described earlier [10]. In brief, for MYB deletion, two predesigned crRNA; Hs. Cas9. MYB.1. AA (MYB exon 6, positive strand, AGG PAM site, sequence: GAATTCTACAATGCGTCGGA) and Hs. Cas9. MYB.1. AB (MYB exon 2, positive strand, TGG PAM site sequence: CAAGTCTGGAAAGCGTCACT) were used to prepare sgRNA. Similarly, for HIF1α deletion, two predesigned crRNA; Hs. Cas9. HIF1A.1. AB (HIF1α exon 3, negative strand, TGG PAM site, sequence: CCTCACACGCAAATAGCTGA) and Hs. Cas9. HIF1A.1. AC (HIF1α exon 6, positive strand, GGG PAM site sequence: ACAGTAACCAACCTCAGTGT) were used. MYB or HIF1α crRNAs were added in an equimolar ratio of Alt-R CRISPR-Cas9 tracrRNA ATTO^™^ 550 and heated at 95 °C for 5 min, thereafter reaction mixture was allowed to cool at RT for 1 h. Cas9 ribonucleoprotein (RNP) complexes were formed by incubating the sgRNA with purified Cas9 Nuclease V3 enzyme (#1081058, IDT) at a 1 μM concentration for 15 min at 37 °C. The Cas9 RNP complexes were then combined with Lipofectamine CRISPRMAX transfection reagent (#CMAX00003, Invitrogen) according to the manufacturer’s instructions. This transfection mixture was added to 3 × 10^5^ cells in 12-well plates. After 48 h, cells were single-cell sorted into 96-well plates based on ATTO^™^ 550 fluorescence and expanded. Positive clones were subsequently confirmed by PCR amplification (data not shown) and Western blotting (Supplementary Fig. S1A and S1B).

### 2.3. Western blot analysis

The cells were processed for Western blot analysis as described earlier by us [19,20]. Briefly, cell lysates were prepared in RIPA buffer supplemented with protease inhibitor cocktail (100 × ). Cell lysates were sonicated (amplitude 20 %, 15 s) to facilitate membrane disruption followed by centrifugation at 10,000 rpm for 10 min. Subcellular fractionation of cell lysates were performed using NE-PER^™^ Nuclear and Cytoplasmic Extraction Reagents (Thermo Fisher Scientific, Logan, UT, USA). Protein quantification was done using DC protein assay kit (Bio-Rad). Total protein (50 μg) was resolved on SDS-PAGE and transferred onto a PVDF membrane (0.45 μm) (Thermo Scientific). Super-Signal West Femto Maximum sensitivity substrate kit (Thermo Scientific) was used to detect protein signal and target protein bands were visualized by ChemiDoc system (Bio-Rad Laboratories, Inc., California, USA).

### 2.4. Confocal microscopy analysis

Cells were cultured in FluoroDish (#FD35–100, World Precision Instruments, Inc., Sarasota, FL, USA) for 48 h. Following media replacement, cells were subjected to either normoxia or hypoxia (1.0 % O_2_, 6 h). Thereafter, cells were washed with 1 × PBS and fixed in 4 % paraformaldehyde for 10 min. After 60 min blocking with 5 % BSA, cells were incubated overnight at 4 °C with a 1:100 dilution of anti-MYB rabbit monoclonal antibody (#12319S, Cell Signaling Technology, Danvers, MA, USA), followed by washing with 1 × PBS and incubation for 60 min with Alexa Fluor 488 anti-rabbit secondary antibody (#A11008, Thermo Scientific, Rockford, IL, USA). After a final wash with 1 × PBS, cells were mounted using Vectashield anti-fade mounting medium (Vector Laboratories, Burlingame, CA, USA) and images were acquired using a Nikon TE2000-E Automated Widefield Microscope equipped with a CoolSnap HQ CCD camera (Nikon Instruments Inc., Melville, NY, USA), as previously described [19].

### 2.5. Chromatin immunoprecipitation (ChIP)

MYB and HIF1α ChIP were carried out using ChIP-IT Express Enzymatic Kit (Active Motif, CA, USA) as previously described [21]. Briefly, cells were cultured in 150 mm dishes for 48 h and subjected to either normoxia or hypoxia (1.0 % O_2_) treatment for 6 h. Following treatment, cells were cross-linked with 1 % formaldehyde for 10 min and the cross-linking was inactivated by 0.125 M glycine for 5 min at room temperature. Cells were then rinsed with ice-cold 1 × PBS and scrapped in ice-cold scraping solution supplemented with protease inhibitor cocktail and PMSF. The collected cells were transferred to ice-cold dounce homogenizer and dounced with appropriate number of strokes to facilitate the nuclei release. Following lysis, cell lysates were centrifuged at 3000×*g* for 10 min in a 4 °C microcentrifuge to pellet the nuclei. The nuclei pellets were resuspended in digestion buffer and enzymatically sheared using enzymatic cocktail provided with the kit. The sheared chromatin was centrifuged at 10,000 rpm for 10 min to collect the supernatant and then subjected to immunoprecipitation reaction overnight at 4 °C using protein G magnetic beads with anti-MYB or anti-HIF1α antibodies. The antibody/protein/DNA complexes were washed with ChIP buffer and cross-linking reversed using reverse cross-linking buffer. The proteins were digested with proteinase K and DNA was purified with QIAGEN PCR column. The purified ChIPed DNA was amplified by PCR using promoter sequence-specific primer sets (Supplementary Table 1).

### 2.6. ChIP-sequencing analysis

Cells were cultured to subconfluence and media was replaced with fresh culture media without antibiotics. Subsequently, cells were subjected to hypoxia (1 % O_2_) in a hypoxia chamber with an oxygen controller for 6 h. Chromatin immunoprecipitation (as described above) was performed in three independent experiments using the antibodies specific to MYB or HIF1α. The purified MYB and HIF1α ChIP DNA samples were submitted for preparation of libraries according to standard protocols. Libraries were sequenced using Illumina NextSeq 500 platforms and the sequencing data analyzed by Basepair software (basepairtech.com). Briefly, the sequencing reads were evaluated for the quality control based on base quality, GC content, presence of sequencing adaptors and overrepresented sequences using FastQC tool. Sequencing reads were then aligned to the Human Reference Genome (GRCh37-hg19 genome assembly) using bowtie and BWA. Quality check was performed for the aligned reads and duplicate reads were removed using Picard tool and uniquely mapped reads were then subjected to peak calling using MACS2 software. The peaks detected in all three replicates with a significance threshold, −log 10 (*p*-value) >5, were included in further analysis. The raw and processed ChIP-sequencing dataset derived from this study was deposited at Gene Expression Omnibus (GEO) database (http://www.ncbi.nlm.nih.gov/geo/) under accession ID GSE292665.

### 2.7. Motif enrichment analysis

HOMER was used for both *de novo* motif discovery and to assess the enrichment of known motifs within 200 bp regions surrounding ChIP-seq peak centers. Motif density histograms for these target regions were generated using HOMER. Control regions, consisting of equal-length DNA sequences located 10 kb downstream of the target regions, were also analyzed. Motif density in the target regions was then normalized to the density in the corresponding control regions.

### 2.8. RNA sequencing analysis

Genetically modified PC cells were cultured under hypoxia for 24 h and total RNA was isolated using QIAGEN RNeasy Mini Kit. RNA samples were submitted for sequencing and bioinformatic analysis at Novogene corporation. RNA quality and quantity was assessed and served as input for poly A^+^ mRNA enrichment. The enriched mRNA was fragmented and reverse transcribed to cDNA. cDNA fragments were then ligated with specific adaptor sequences and used for library preparation. The cDNA library was sequenced using the Illumina NovaSeq 6000 platform. Raw sequencing reads underwent quality control check, and paired-end clean reads were aligned to human hg19 reference genome using TopHat 2 software. Differential gene expression analysis was performed using the DESeq2 R package (version 1.30.1). Volcano plots and heatmaps of differentially expressed genes were generated using the SRplot online tool [22]. The RNA-seq data in this study was deposited at GEO database under accession ID GSE292666.

### 2.9. Pathway enrichment analysis

KEGG pathway enrichment analysis for differentially expressed genes was performed using Enrichr (https://maayanlab.cloud/Enrichr) [23]. Gene Set Enrichment Analysis (GSEA) of differentially expressed genes was performed using the GSEA software [24]. Hallmark gene set was downloaded from MSigDB databases and used to identify enriched pathways. A weighted enrichment score (ES) was calculated to determine whether genes within a predefined gene set were significantly enriched at the top or bottom of the ranked gene list. Nominal p-values and normalized enrichment scores (NES) were generated to assess the statistical significance of the enrichment. False discovery rate (FDR) was used to correct for multiple testing.

### 2.10. Gene expression analysis and correlation with tumor grade and patient survival

The differential gene expression between normal and pancreatic cancer, and the association of gene expression levels with patient survival were analyzed using Gepia2 platform [25]. The human protein atlas (HPA) database was also queried to examine the differential expression at the protein level. The correlation of gene expression with tumor grade was analyzed on UALCAN platform using the TCGA database [26]. Grade 4 tumors were excluded from the study due to their very small sample size (n = 2).

### 2.11. Statistical analysis

Each RNA-seq and ChIP-seq experiment was performed in three independent biological replicates. For ChIP-seq analysis, peaks were identified relative to input controls to correct for background signal, and only peaks with a statistical significance of −log_10_ (*p*-value) > 5 were included in downstream analyses. For RNA-seq, differentially expressed genes (DEGs) were defined as those showing a fold change (FC) > 1.5 and a *p*-value <0.05. For all other experiments, statistical significance was calculated using Prism 8.1 (GraphPad) spreadsheets by employing either student’s t-test to compare two groups, or one-way ANOVA for multiple comparisons. Experiments were performed at least three times, and data was expressed as mean ± SD. A *p*-value of <0.05 was considered statistically significant.

## 3. Results

### 3.1. MYB exhibits altered genomic occupancy under hypoxia

**Fig. 1.**
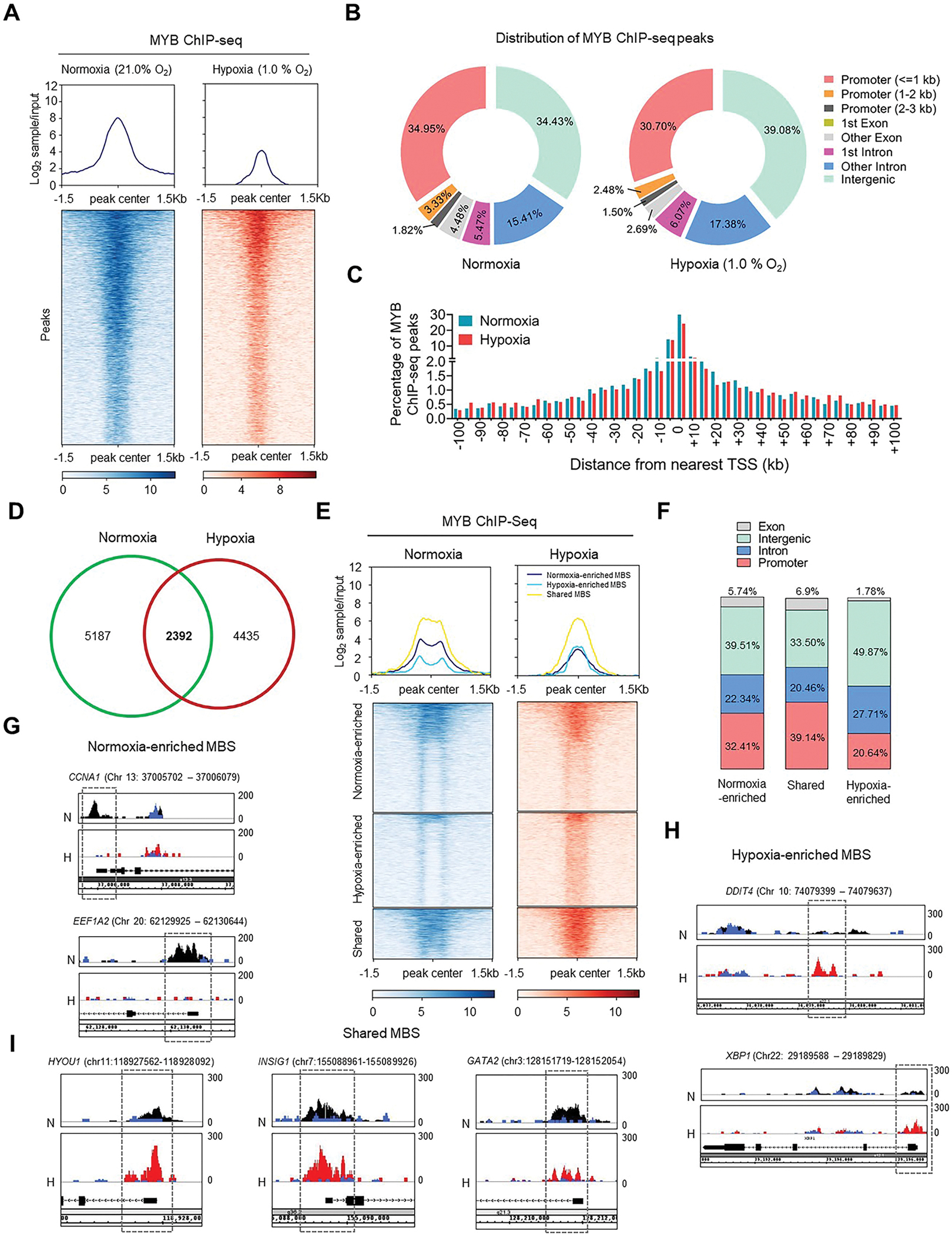
Genomic occupancy of MYB is altered under hypoxia: (**A**) Differential binding of MYB to genomic sequences under normoxia and hypoxia (1.0 % O_2_, 6 h) was examined by performing ChIP-seq analysis of MiaPaCa cells in three biological replicates. Line plots and heatmaps were prepared from MYB ChIP-seq peaks after normalization with respective input controls using DeepSeq tools. Upper panels show the average MYB ChIP-seq signal intensities and lower panels show density heatmaps depicting MYB peaks enrichment around peak center under normoxia and hypoxia conditions. (**B**) Pie charts show differential distribution of MYB peaks across the defined genomic regions under normoxia and hypoxia. MYB ChIP-seq peaks with significance threshold of −log_10_ (*p-*value >5) from three replicates were included in the analysis as described in the Methods section. (**C**) Bar graph shows the percentage of MYB ChIP-seq peaks under normoxia (sky blue bars) and hypoxia (red bars) present within the ±100 kb region from the nearest TSS at ±5 kb bin intervals prepared from MYB ChIP-seq analyses. (**D**) Unique and overlapped genomic binding sites of MYB are presented in the venn diagram after comparing the MYB ChIP-seq profiles under normoxia and hypoxia. (**E**) MYB binding sites were categorized into normoxia-enriched, shared, and hypoxia-enriched sites based on comparative analysis of MYB ChIP-seq peaks under normoxia (left) and hypoxia (right). The upper panels show the normalized ChIP-seq signal intensities of enriched and shared MYB binding sites under normoxia and hypoxia. Heatmap view (lower panel) of MYB ChIP-Seq reads intensity around peak center (±1.5 kb) identified in ChIP-seq analyses under normoxia and hypoxia was divided into normoxia-enriched, hypoxia-enriched, and shared sites. (**F**) Percent distribution of normoxia-enriched sites, shared sites and hypoxia-enriched sites across the defined genomic regions. (**G-I**) Integrated Genome Browser (IGB) visualization of MYB peaks enrichment under normoxia (N, track shown in black) and hypoxia (H, track shown in red) at promoter or intergenic region of various putative target genes. IGB view of normoxia-enriched MYB binding on representative genes (*CCNA1*, and *EEF1A2*) show enrichment of MYB ChIP-seq signal exclusive to normoxia (**G**). Genomic visualization of hypoxia-enriched MYB binding sites showed significantly greater enrichment of MYB binding on DDIT4, and XBP1 genes under hypoxia compared to normoxia (**H**). Visualization of representative shared sites showing increased (*HYOU1* and *INSIG1*) and decreased (*GATA2*) enrichment of MYB binding peaks under hypoxia (**I**). The respective input control tracks with no enrichment of MYB binding are shown in blue color. (For interpretation of the references to color in this figure legend, the reader is referred to the Web version of this article.)

To investigate the effect of hypoxia on MYB genomic occupancy, we performed ChIP-seq analysis in pancreatic cancer cells grown under hypoxia and normoxia. Comparison of ChIP-seq read intensity heat map revealed a more focused and less intense peaks under hypoxia compared to normoxia suggesting a genome-wide reduction in MYB binding (Fig. 1A). MYB peaks decreased under hypoxia across all the genomic regions with highest reduction observed in the promoter and intergenic regions (Supplementary Fig. S2A). This decrease in MYB genomic binding under hypoxia did not result from its altered subcellular localization (Supplementary Fig. S2B and S2C). Further, despite the reduction in the number of MYB peaks, peak scores were largely similar under both normoxia and hypoxia (Supplementary Fig. S2D). The genomic distribution profile of ChIP-seq peaks revealed predominant binding of MYB in the promoter ( ≤ 1 kb) and intergenic regions followed by intronic sequences under normoxia, whereas the major proportion of MYB peaks were located in the intergenic region followed by promoters and introns under hypoxia (Fig. 1B and C). A biotype distribution profile of MYB-binding sites (MBS) indicated a slightly greater distribution for the non-coding RNA gene targets and lesser for the protein-coding gene targets under hypoxia (Supplementary Fig. S2E). Comparison of MYB ChIP-seq profiles between normoxia and hypoxia revealed that 5187 MBS were lost under hypoxia along with a gain of 4435 new sites. 2392 MBS were overlapping under both conditions (Fig. 1D). Overlapped MBS had the strongest ChIP-seq read intensity, suggesting high-affinity chromatin interaction, whereas hypoxia-enriched sites had lesser peak enrichment than the normoxia-enriched MBS (Fig. 1E). Genomic distribution of these unique and overlapping MBS was also different with ~32 %, ~39 %, and ~20 % of normoxia-enriched, shared, and hypoxia-enriched sites being in the promoter regions, respectively. In contrast, about 50 % of hypoxia-enriched sites were present in the intergenic regions, compared to ~39 % and ~33 % of normoxia-enriched and shared sites, respectively (Fig. 1F).

Visualization of representative MYB peaks using the integrated genome browser (IGB) shows exclusive enrichment of MBS in the promoter regions of *CCNA1* and *EEF1A2* under normoxia, whereas MBS were enriched in *DDIT4* (intergenic region) and *XBP1* promoter region under hypoxia (Fig. 1G and H). Shared MBS (*HYOU1*, *INSIG1* and *GATA2*) revealed similar or somewhat differential enrichment under both the conditions (Fig. 1I). This redistribution of MYB binding does not appear to be cell line-specific, since hypoxia exposure of other cell lines (Panc-1 and BxPC3) recapitulates the differential binding of MYB on these representative targets, resulting in both stronger and weaker MYB-binding events (Supplementary Fig. S2F). Since shared sites showed strong peak enrichment under both conditions, we examined if MYB binding increased or decreased in those regions under hypoxia. Approximately 20 % of these shared sites showed increased binding under hypoxia whereas it decreased (~38 %) or remained unchanged (~42 %) in the remaining regions (Supplementary Fig. S2G). Interestingly, hypoxia-enriched or unchanged shared sites are predominantly bound to the promoter region (61.02 % or 55.66 %, respectively), whereas hypoxia-decreased sites had predominant distribution (43.43 %) in the intergenic regions (Supplementary Fig. S2H). These findings suggest an altered genomic occupancy of MYB under the low-oxygen environment.

### 3.2. Altered genomic occupancy of MYB under hypoxia is partly dependent on HIF1α

**Fig. 2.**
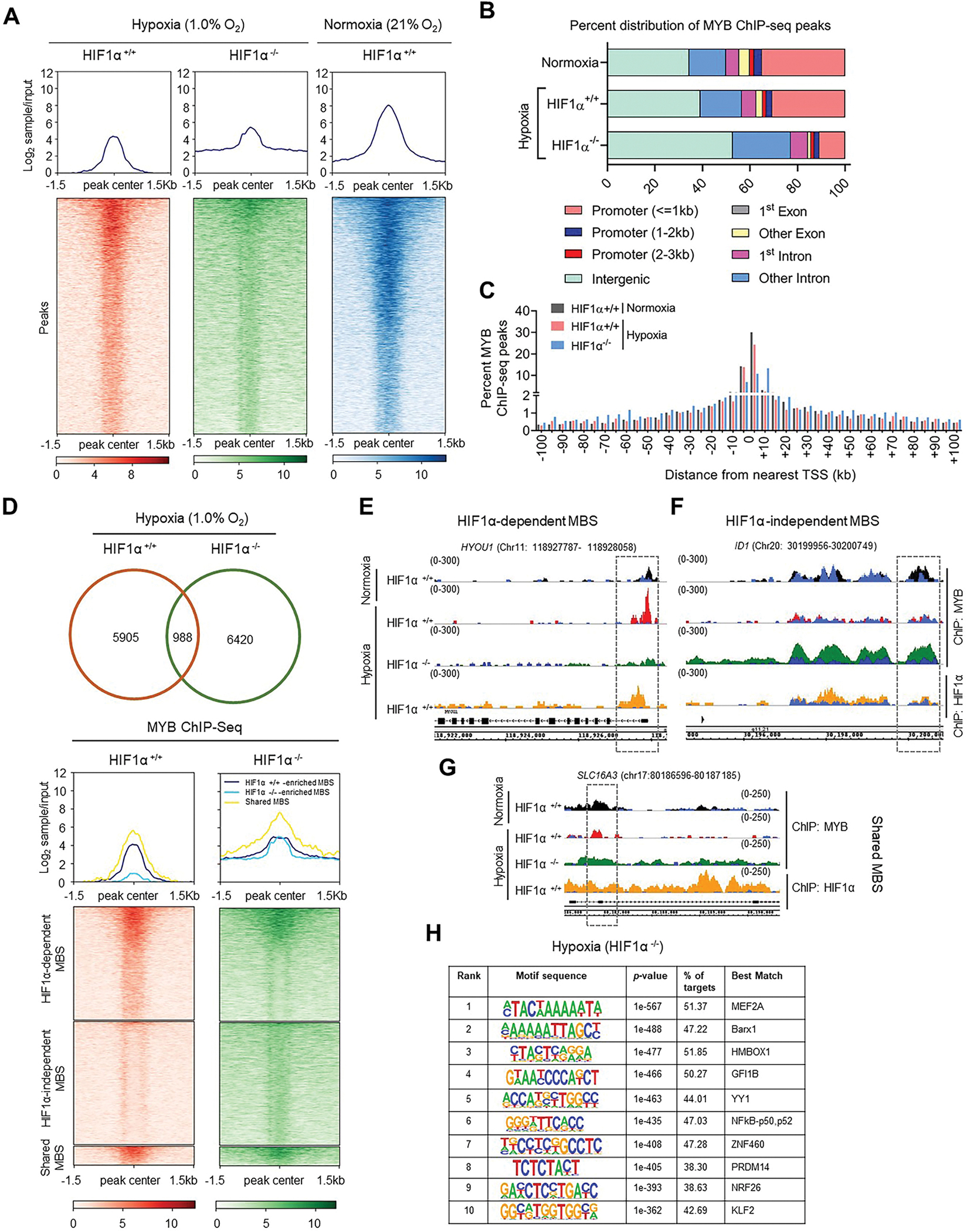
Dependency of differential chromatin occupancy of MYB under hypoxia on HIF1α: (**A**) MYB ChIP-seq analysis was performed in HIF1α knockout (HIF1α ^−/−^ ) cells exposed under hypoxia and compared to MYB ChIP-seq profiles conducted in control (HIF1α ^+/+^) cells under both hypoxia and normoxia conditions. Average line plots depict the MYB ChIP-seq signal intensities in HIF1α ^+/+^ and HIF1α ^−/−^ cells (upper panels). Density heatmaps showing the enrichment of ChIP-seq peaks near ±1.5 kb of peak center (lower panels). (**B**) Comparison of genomic distribution of MYB ChIP-seq peaks (−log_10_ (*p-*value >5), three replicates) in HIF1α ^−/−^ cells (under hypoxia only) and HIF1α ^+/+^ cells (under both hypoxia and normoxia) at defined genomic regions. (**C**) Bar diagram showing the percentage of MYB peaks in HIF1α ^+/+^ cells under normoxia (gray bars) or hypoxia (red bars) and HIF1α ^−/−^ cells under hypoxia only (blue bars) at ±5 kb bin intervals from the nearest TSS. (**D**) Venn diagram depicting the comparison of MYB ChIP-seq peaks identified in HIF1α ^+/+^ and HIF1α ^−/−^ cells (upper panel). Density heatmap view of MYB binding sites around peak center (±1.5 kb) detected in HIF1α^+/+^ and HIF1α^−/−^ cells under hypoxia. MBS were separated into HIF1α-dependent (enriched in HIF1α^+/+^ cells) or HIF1α-independent sites (enriched in HIF1α^−/−^ cells) or shared sites (enriched in both HIF1α^+/+^ and HIF1α^−/−^ cells). (**E-G**) IGB visualization of MYB peak enrichment in HIF1α ^+/+^ cells under normoxia (black track) and hypoxia (red track) and in HIF1α ^−/−^ cells (green track) under hypoxia at chromosomal regions of representative HIF1α-dependent MYB binding sites (**E**), HIF1α-independent sites (**F**) and shared sites (**G**). HIF1α binding in HIF1α ^+/+^ cells was also detected near these sites and represented as golden track. The respective input control tracks with poor enrichment of MYB binding are shown in blue color. (**H**) *de novo* motif co-occurrence of various transcription factors near MYB binding sites under hypoxia. (For interpretation of the references to color in this figure legend, the reader is referred to the Web version of this article.)

Since HIF1α is a master regulator of hypoxia adaptive response and we previously observed MYB-HIF1α interaction, we asked if it played a role in altered genomic occupancy of MYB. MYB ChIP-Seq was performed in the presence or absence of HIF1α using the control and knockout cell lines. The data showed a widely dispersed MYB binding from the peak center in HIF1α^−/−^ cells under hypoxia, along with a slight increase in MYB enrichment, which is partly similar to MYB ChIP-seq profile under normoxia (Fig. 2A). MYB expression remained largely unchanged in HIF1α^−/−^ and HIF1α^+/+^ cells (Supplementary Fig. S3A). Analysis of genome-wide distribution of MYB peaks in HIF1α^+/+^ and HIF1α^−/−^ cells revealed a shift in MBS from promoter ( ≤ 1 kb) to other intron and intergenic regions. This redistribution in MYB peaks, however, was completely different than the genomic distribution observed in HIF1α^+/+^ cells under normoxia (Fig. 2B and Supplementary Fig. S3B) suggesting that MYB genomic occupancy is altered through complex nuclear interactions. Interestingly, most frequent MYB binding was observed within the ±5 kb region around the transcriptional start site (TSS) in HIF1α^+/+^ cells under normoxia and hypoxia, whereas it reduced in this region in HIF1α^−/−^ cells and exhibited a concomitant increase in the distal regions (Fig. 2C). Also, MYB peaks in the absence of HIF1α showed a slightly reduced distribution in the protein-coding genes shifting towards noncoding RNA targets (Supplementary Fig. S3C).

Deletion of HIF1α led to a loss of MYB binding on 5905 sites (HIF1α-dependent, enriched in HIF1α^+/+^ cells) under hypoxia, while 6420 new sites (HIF1α-independent, enriched in HIF1α^−/−^ cells) were gained. MYB binding was retained on 988 sites (shared sites, enriched in both HIF1α^+/+^ and HIF1α^−/−^ cells) (Fig. 2D). Of 7408 MYB binding sites (MBS) retained and/or gained under hypoxia in HIF1α^−/−^ cells, 4737 (~64 %) were shared with MBS in HIF1α^+/+^ cells under normoxia, suggesting a significant role of HIF1α in altered genomic occupancy of MYB under hypoxia (Supplementary Fig. S3D). Genomic visualization of HIF1α-dependent MBS in a randomly selected gene, *HYOU1,* shows greater enrichment of MYB peak in the promoter region under hypoxia (red track) than under normoxia (black track), which was diminished upon deletion of HIF1α (green track). We also observed HIF1α peak enrichment (golden track) in this genomic region (Fig. 2E). HIF1α-independent MBS in another randomly picked gene, *ID1*, shows MYB peak enrichment under normoxia (in absence of HIF1 α accumulation) and in HIF1α-deleted cells subjected to hypoxia, without HIF1α peak enrichment in HIF1α^+/+^ cells within that region (Fig. 2F). *SLC16A3* represents a shared MBS that does not exhibit marked alteration in MYB peak enrichment in HIF1α^+/+^ and HIF1α^−/−^ cells under hypoxia (Fig. 2G). We further confirmed a similar MYB binding pattern in additional genetically engineered pancreatic cancer cell lines (Supplementary Fig. S3E). These results suggest that MYB binding to a subset of genomic regions is influenced by HIF1α under hypoxia. Interestingly, MYB binding under hypoxia were predominantly located in the promoter region in the presence of HIF1α and got shifted to the intronic and intergenic regions in HIF1α^−/−^ cells. Shared sites showed equal distribution in the promoter and intergenic regions with some gain in the intronic regions (Supplementary Fig. S3F). Since HIF1α seemed to be partly responsible to altered MYB genomic occupancy, we performed *de novo* motif enrichment analysis near MBS. We identified a range of transcriptional factors, including NFkB-p50 and YY1, which are reported to have HIF1α-independent role in hypoxia adaption, suggesting their potential involvement in altered genomic occupancy of MYB or *vice versa* (Fig. 2H).

### 3.3. MYB has a significant impact on the genomic distribution of HIF1α

**Fig. 3.**
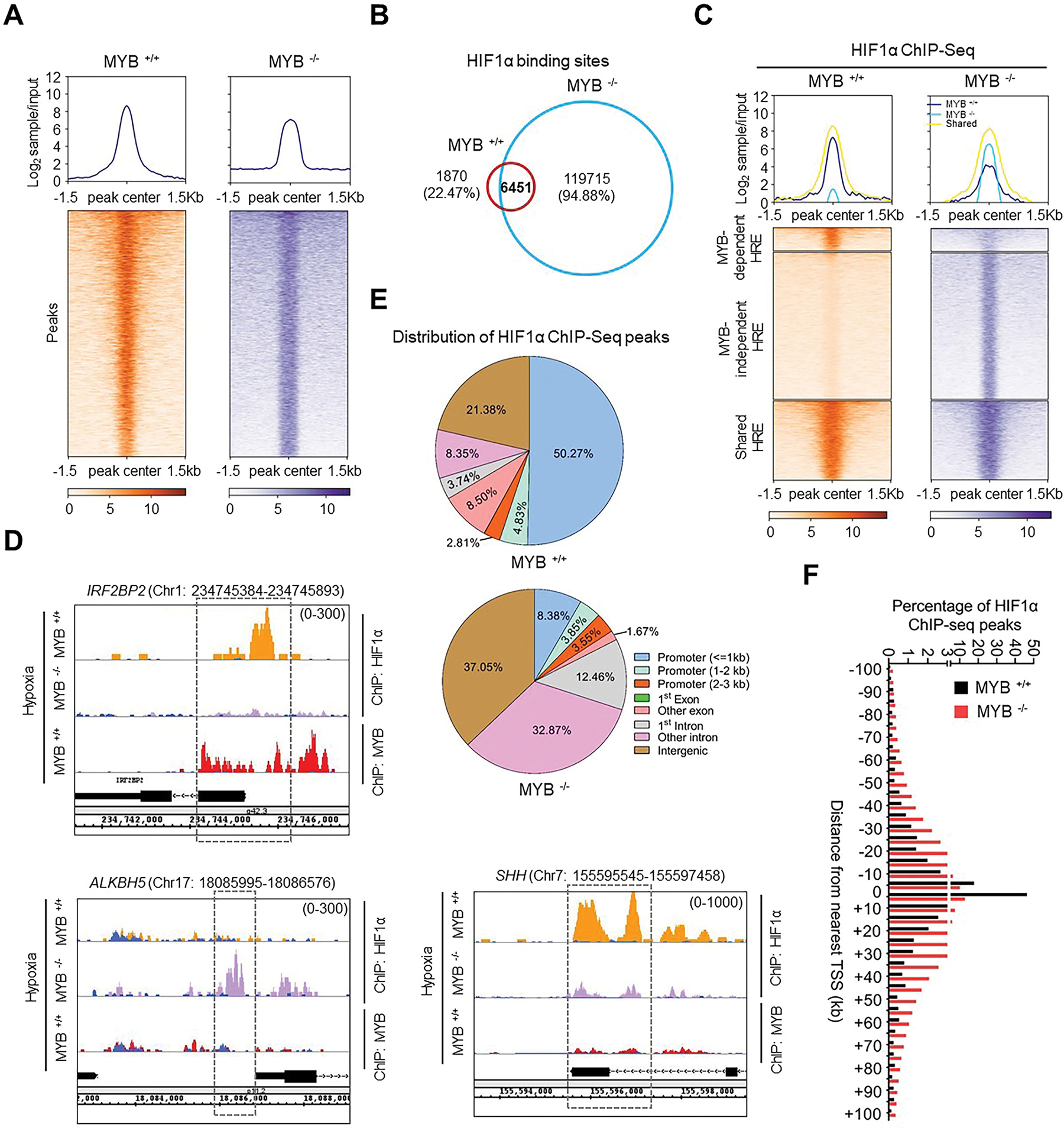
MYB affects the binding of HIF1α at proximal promoter regions: (**A**) The effect of MYB deletion on genomic occupancy of HIF1α was analyzed by comparing the HIF1α ChIP-seq profiles in control (MYB ^+/+^) and MYB knockout cells with ectopic expression of HIF1α (MYB ^−/−^). The average line plot shows the input normalized HIF1α ChIP-seq signal intensities in MYB ^+/+^ and MYB ^−/−^ cells (upper panels). Heatmap display aggregation of HIF1α ChIP-seq peaks enrichment at ± 1.5 kb distance from peak center. (**B**) Comparison of HIF1α ChIP-seq peaks (−log_10_ (*p-*value >5), from three replicates) presented in venn diagram shows overlapped and unique HIF1α-bound genomic regions between MYB ^+/+^ and MYB ^−/−^ cells. (**C**) HIF1α ChIP-seq peaks were divided into three categories: MYB-dependent HRE (enriched in MYB^+/+^ cells), MYB-independent HRE (enriched in MYB^−/−^ cells), and shared HRE (enriched in both MYB^+/+^ and MYB^−/−^ cells). The upper panel shows the average line plot depicting the normalized signal intensities in each category in MYB ^+/+^ and MYB ^−/−^ cells. The lower panel shows the density heatmap of HIF1α peaks enrichment near peak center (±1.5 kb) in these respective categories. (**D**) IGB visualization of HIF1α peaks in MYB ^+/+^ (golden track) and MYB ^−/−^ cells (purple track) at the promoter regions of respective sites (*IRF2BP2*, *ALKBH5*, and *SHH*). The respective input control tracks are shown in blue color. (**E**) Pie charts depict the distribution of HIF1α ChIP-seq peaks across the defined genomic regions in MYB ^+/+^ (upper panel) and MYB ^−/−^ cells (lower panel). (**F**) Bar graph shows percent HIF1α binding sites within ±100 kb region from nearest TSS at ± 5 kb bin interval as analyzed from HIF1α ChIP-seq profiles in MYB ^+/+^ (black bars) and MYB ^−/−^ cells (red bars). (For interpretation of the references to color in this figure legend, the reader is referred to the Web version of this article.)

Having observed an effect of HIF1α on MYB genomic occupancy under hypoxic environment, we asked if MYB also impacts the genomic distribution of HIF1α. For this, we utilized MYB-knockout (MYB^−/−^ ) cells with ectopically restored HIF1α expression level generated as part of our previous study [10]. Expression levels of MYB and HIF1α were confirmed by immunoblotting (Supplementary Fig. S4A). MYB^−/−^ cells and respective MYB-expressing control (MYB^+/+^) cells were cultured under hypoxia for 6 h and subjected to HIF1α ChIP-seq. Analysis of ChIP-seq peak density heatmaps revealed a more dispersed peaks and substantially decreased HIF1α ChIP-seq read intensity in MYB^−/−^ cells compared to MYB^+/+^ cells (Fig. 3A). Unexpectedly, we observed an overall increase in total binding events of HIF1α in MYB^−/−^ cells across all the defined genomic regions (Supplementary Fig. S4B). This large increase in HIF1α binding events led to a ~5-fold increase in the average number of peaks assigned to their corresponding gene targets (Supplementary Fig. S4C). While a large majority of peaks (~78 %; 6451 peaks) were retained (shared hypoxia-responsive elements, HRE) after MYB deletion, twice as many new HIF1α ChIP-seq peaks (119715 peaks) were gained (MYB-independent HRE, enriched in MYB^−/−^ cells) and a small fraction (~22 %, 1870 peaks) (MYB-dependent HRE, enriched in MYB^+/+^ cells) were lost (Fig. 3B). Shared sites had the highest enrichment of HIF1α, followed by MYB-dependent HRE, while a comparable enrichment of HIF1α on MYB-independent HRE was also reported in MYB^−/−^ cells (Fig. 3C). Co-occupancy of MYB and HIF1α with high enrichment was reported for shared and MYB-dependent sites, whereas it was not the case for MYB-independent HREs (Fig. 3D). This binding pattern of HIF1α and MYB was confirmed by ChIP-qPCR assay on select sites in additional cancer cell lines (Supplementary Fig. S4D). HIF1α predominantly bound to the proximal promoters ( ≤ 1 kb) (~50 %), which shifted more towards intronic (from ~12 % to ~43 %) and intergenic (from ~21 % to ~37 %) regions with only ~8 % binding in the promoter region upon MYB deletion (Fig. 3E). A higher binding of HIF1α was reported near the TSS within the ±5 kb region, in presence of MYB, which shifted toward a higher binding in the far regions (>±5 kb) of TSS in the absence of MYB (Fig. 3F). Together, these findings suggest a significant role of MYB in defining the HIF1α binding preference under hypoxia.

### 3.4. MYB plays a significant role in hypoxia-induced transcriptional reprogramming

**Fig. 4.**
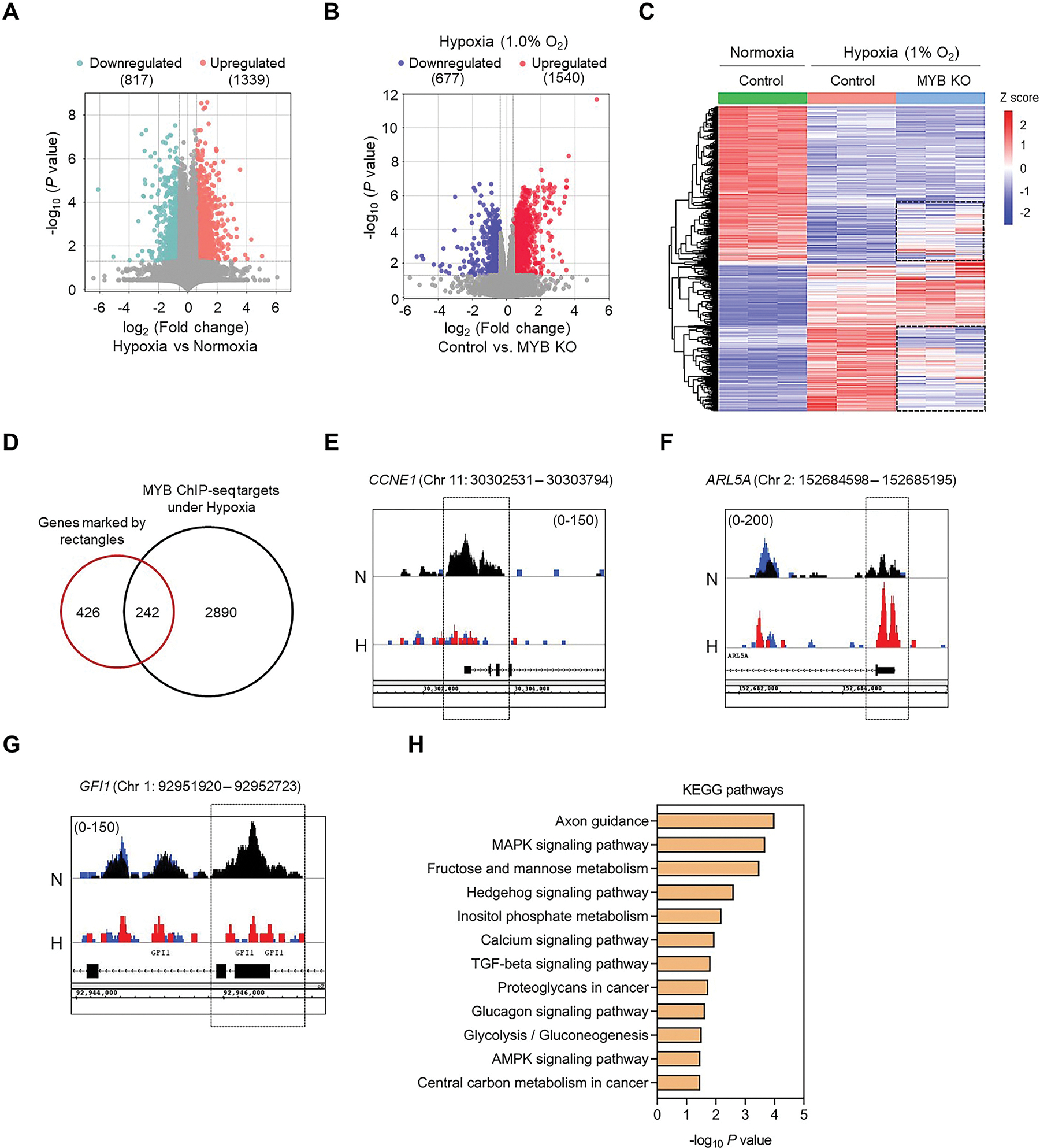
Role of MYB-dependent transcriptional output in hypoxia adaptive response: (**A**) A volcano plot illustrating the differentially expressed genes (DEGs) obtained from RNA-seq analysis of hypoxia-treated pancreatic cancer cells compared to normoxia. Hypoxia-induced upregulated and downregulated genes are shown in orange color and turquoise color respectively. (**B**) RNA-seq analysis was performed to determine the DEGs under hypoxia in MiaPaCa cells following MYB deletion. The volcano plot shows the differentially expressed genes from RNA-seq analysis when control cells were compared with MYB KO cells under hypoxia. The genes upregulated and downregulated in control cells shown by red color and blue color respectively. (**C**) Heatmap of RNA-seq data showing the k-means clustering of significant DEGs affected by hypoxia in control and MYB knockout cells compared to control cells under normoxia. A subset of genes that failed to be properly altered under hypoxia in MYB knockout cells are marked by rectangular boxes. (**D**) Venn diagram depicts the comparison of putative MYB ChIP-seq targets (protein coding genes) under hypoxia with the genes not sufficiently altered under hypoxia (marked by rectangle) in MYB KO cells. Integrated Genome Browser (IGB) software was used to visualize MYB binding sites on the promoter region of *CCNE1* (**E**), *ARL5A* (**F**), and the exonic region of *GFI1* (**G**) under normoxia and hypoxia conditions. N-Normoxia (black track), H-Hypoxia (red track); The input control track is shown in blue color. (**H**) Bar diagram showing the enriched KEGG pathways associated with direct targets of MYB that were not altered properly under hypoxia. The vertical axis shows the pathways terms, and the horizontal axis depicts the −log_10_ *p-*value of individual pathway. (For interpretation of the references to color in this figure legend, the reader is referred to the Web version of this article.)

RNA-sequencing (RNA-seq) of MYB-expressing pancreatic cancer cells under normoxia (21 % O_2_) and hypoxia (1.0 % O_2_) identified 2156 differentially expressed genes (DEGs) (fold change ≥1.5, *p* value < 0.05), of which 1339 were upregulated and 817 downregulated in hypoxia-exposed cells (Fig. 4A). These changes were associated with gain of glycolytic metabolism, epithelial-mesenchymal transition (EMT), survival, migration and angiogenic phenotypes, whereas genes associated with cell cycle progression, DNA repair, ribosome biogenesis, and oxidative phosphorylation were suppressed (Supplementary Fig. S5A–S5C). To analyze the role of MYB in this transcriptional reprograming associated with hypoxia adaptive response, we performed RNAseq analysis on MYB-overexpressing and -knockout cells cultured under hypoxia. A total of 2217 DEGs (fold change ≥1.5, *p* value < 0.05) were identified, of which 1540 were upregulated and 677 downregulated in MYB KO cells (Fig. 4B). The comparison of MYB-dependent DEGs under hypoxia with hypoxia-induced DEGs identified a subset of hypoxia-responsive genes, whose induction or repression was dependent on MYB expression (Fig. 4C). Further, integrative analysis of these MYB-dependent hypoxia regulated targets (marked by rectangles) with MYB ChIP-seq putative targets (protein coding only) revealed a total of 242 targets out of 668 were likely the targets of MYB under hypoxia (Fig. 4D). Among these, there were several established targets of MYB, such as *CCNE1* [27,28], *ARL5A* [29], and *GFI1* [30], which also exhibited altered MYB binding in their regulatory regions under hypoxia (Fig. 4E–G). Further, KEGG pathway enrichment analysis of these MYB direct targets under hypoxia showed the involvement of MYB in axon guidance, several oncogenic signaling (MAPK, hedgehog, TGFβ, AMPK) pathways, calcium signaling, glucagon signaling pathway and metabolic pathways (Fig. 4H). These results suggest an important role of MYB in several signaling pathways involved in hypoxia adaptive response.

### 3.5. MYB and HIF1α co-regulated genes are associated with hypoxia adaptive signaling pathways, tumor progression, and poor survival of pancreatic cancer patients

**Fig. 5.**
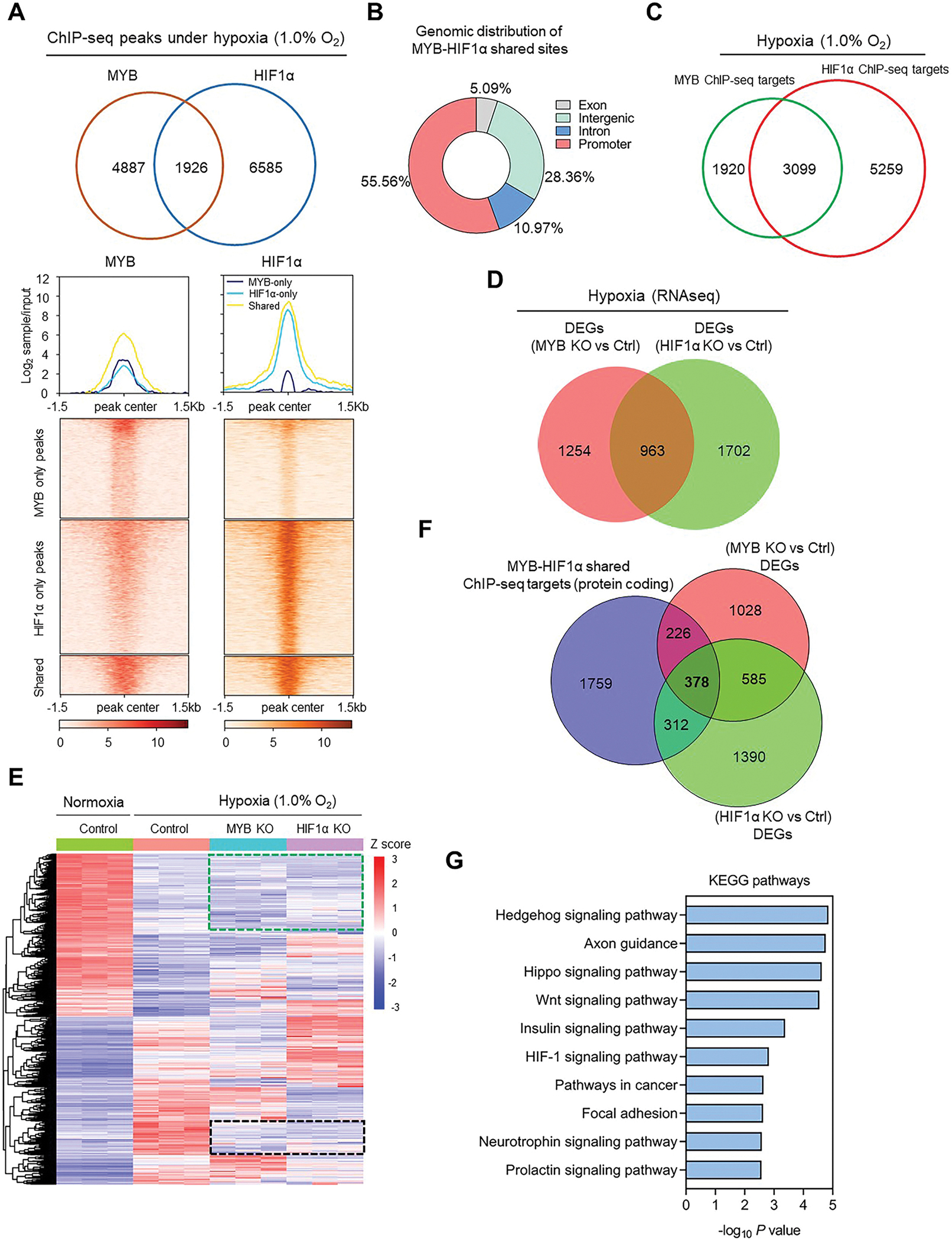
Role of MYB-HIF1α-shared transcriptional output in hypoxia adaptive response: (**A**) Venn diagram shows the comparison of MYB and HIF1α ChIP-seq profiles. MYB and HIF1α peaks were divided into three groups: MYB only, HIF1α only, and shared sites. The upper panel shows the line plot depicting the input normalized ChIP-seq signal intensities in each group. The density heatmap shows the MYB and HIF1α peak enrichment near peak center in all three categories (lower panel). (**B**) Distribution of MYB-HIF1α-shared sites across the defined genomic regions. (**C**) Venn diagram depicting the MYB and HIF1α-overlapped and unique ChIP-seq putative targets. (**D**) Comparison (overlapped and unique) of DEGs identified from RNA-seq analyses of MYB KO vs ctrl (red color) and HIF1α KO vs ctrl (green color). (**E**) Heatmap analysis of RNA-seq depicting the k-means clustering of significant DEGs affected by hypoxia in control, MYB knockout and HIF1α knockout cells compared to control cells under normoxia. A subset of genes that failed to be induced under hypoxia in both MYB KO and HIF1α KO cells as marked by black rectangular box. Green rectangle box shows the fraction of genes that were not affected with either MYB and HIF1α. (**F**) Venn diagram detailing the genes overlap among DEGs from MYB- and HIF1α-depleted cells and MYB-HIF1α overlapped putative ChIP-seq target genes. (**G**) KEGG pathway enrichment analysis of direct gene targets overlapped between MYB and HIF1α was examined using Enrichr. (For interpretation of the references to color in this figure legend, the reader is referred to the Web version of this article.)

**Fig. 6.**
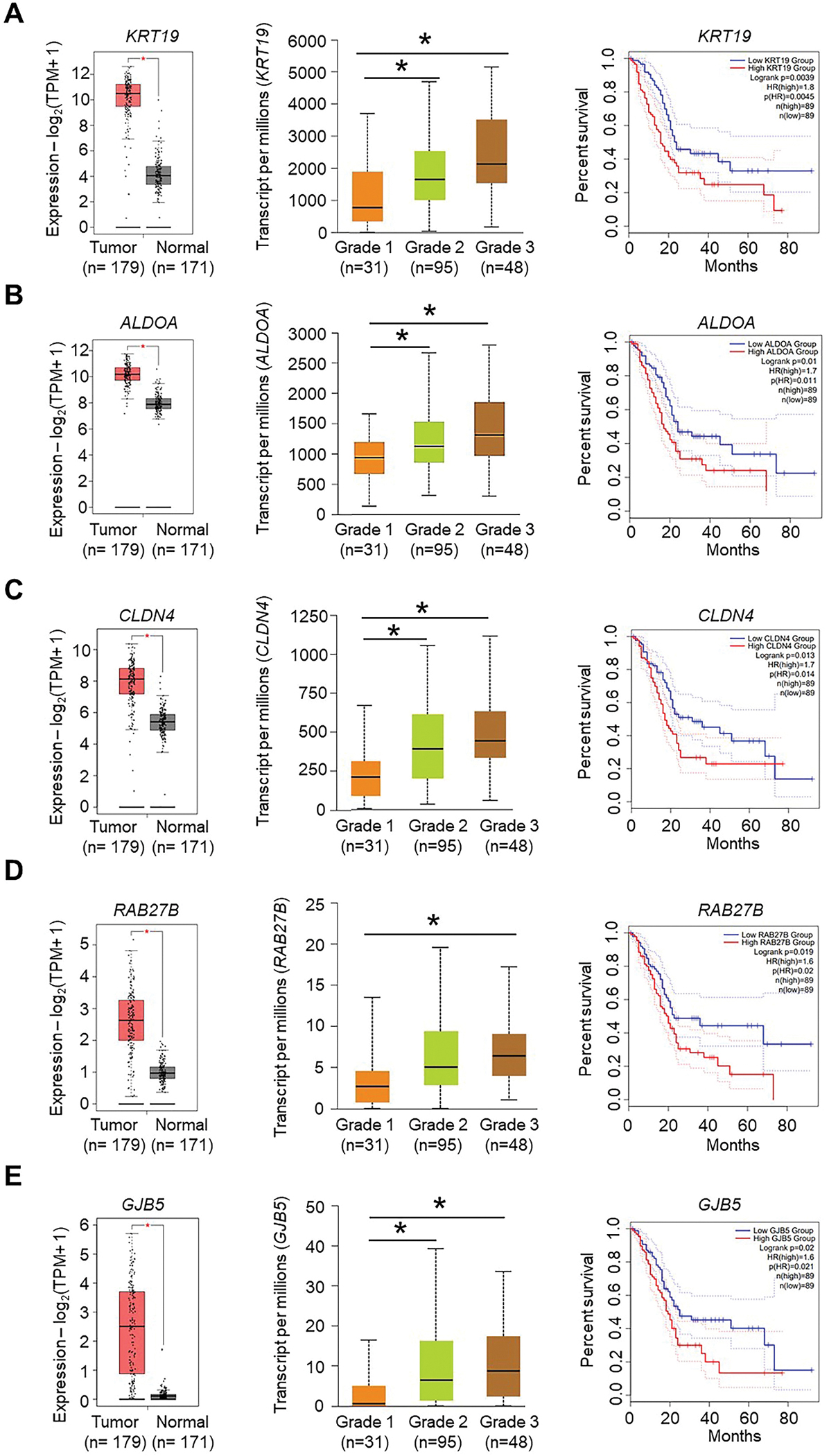
Expression of *KRT19*, *ALDOA*, *CLDN4*, *RAB27B*, and *GJB5* in pancreatic cancer patients and association of their expression with tumor grade and patients’ survival. **(A**–**E)** The mRNA expression of *KRT19*, *ALDOA*, *CLDN4*, *RAB27B*, and *GJB5* was examined in pancreatic cancer patients (combined TCGA and GTEx database) using Gepia2. Clinicopathologic association of the expression of *KRT19*, *ALDOA*, *CLDN4*, *RAB27B*, and *GJB5* with tumor grade and patient’s survival was analyzed using UALCAN software.

To understand the functional relevance of MYB-HIF1α crosstalk in the hypoxia adaptive response, we first compared HIF1α and MYB ChIP-seq data to identify their shared binding sites. Approximately 28 % of total MYB binding events overlapped with HIF1α binding sites, indicating their potential interaction with gene regulatory consequences (Fig. 5A). Genomic distribution indicated a greater proportion of MYB and HIF1α-overlapped sites in promoters followed by intergenic regions (Fig. 5B). We then identified putative MYB and HIF1α gene targets being regulated through these binding events using HOMER algorithm. Analysis of these putative MYB and HIF1α targets (including coding and noncoding) revealed 3099 shared targets (~62 % of total identified MYB ChIP-seq targets) (Fig. 5C). About ~9.6 % of these shared ChIP-seq targets were among those induced under hypoxia, compared to 4.5 % and 6.4 % targets unique to MYB and HIF1α, respectively (Supplementary Fig. S6A). RNAseq analysis of HIF1α knockout cells (HIF1α KO vs Ctrl) under hypoxia showed 2665 significantly DEGs (fold change ≥1.5, *p* < 0.05) in HIF1α-deleted MiaPaCa cells (Supplementary Fig. S6B). Of these, 963 were overlapped with MYB-dependent DEGs (Fig. 5D). Comparison of MYB- and HIF1α-dependent DEGs with hypoxia-induced DEGs identified a subset of hypoxia-responsive genes, whose induction was abrogated upon deletion of either MYB or HIF1α (marked by black rectangle). Interestingly, a large fraction of genes (marked by green rectangle) remained unaltered even after MYB or HIF1α deletion, suggesting the involvement of additional gene regulator (s) (Fig. 5E). Integration analysis of MYB-HIF1α-shared ChIP-seq targets with MYB- and HIF1α-dependent overlapping genes identified a total of 378 genes (309 downregulated and 69 upregulated) as shared direct targets with functional consequences (Fig. 5F and Supplementary Fig. S6C). KEGG pathway analysis of these genes underscored the cooperative/collaborative role of MYB and HIF1α in several oncogenic signaling pathways, including Hedgehog signaling, Axon guidance, Hippo signaling, Wnt signaling, Insulin signaling, Focal adhesion, Neurotrophin and Prolactin signaling pathways (Fig. 5G). Furthermore, five genes (*KRT19*, *ALDOA*, *CLDN4*, *RAB27B*, and *GJB5*) among the top 10 % coregulated genes were identified to have significantly elevated expression in pancreatic cancer compared to the normal pancreas (Fig. 6A–E, Supplementary Fig. S7A–S7E). Moreover, expression of these genes exhibited a significant positive association with increasing tumor grade and correlated inversely with pancreatic cancer patients’ survival (Fig. 6A–E). These observations suggest a crucial role of MYB-HIF1α crosstalk in optimal adaptation of pancreatic cancer cells to hypoxic stress and gain of aggressive tumor phenotypes to support relentless progression of pancreatic cancer.

## 4. Discussion

This study examined the role of MYB in hypoxia-induced transcriptional reprogramming and associated adaptive signaling alterations. We observed that the genomic occupancy of MYB is significantly altered under hypoxia, in part, through its interaction with HIF1α. More importantly, we observed that MYB also had a huge impact on the genomic occupancy of HIF1α. Finally, our data revealed the functional implications of this interaction in shaping the transcriptional landscape to optimally support the hypoxic adaptive response. Genes coregulated through MYB-HIF1α crosstalk exhibited increased expression in clinical specimens and correlated positively with disease progression and poor patient survival.

Hypoxia is an inevitable stress for all solid tumors, and the tumor cells must adapt to this restrictive TME pressure to sustain their progressive journey [31,32]. Our previous work demonstrated a significant role of MYB in supporting the hypoxic survival of pancreatic cancer cells through its induced expression and metabolic reprogramming in cooperation with HIF1α [10]. Here we observed a significant redistribution of MYB binding across the genome under hypoxia, characterized by a decrease at promoters and an increase in the intergenic regions. Decrease on MYB peak intensity suggested reduced genome-wide occupancy, however, peak scores remained relatively constant suggesting that the binding affinity of MYB towards its hypoxia-specific gene targets remained unchanged. This implies a more selective recruitment of MYB to specific genomic regions under hypoxia, potentially driven by altered chromatin accessibility under hypoxic tumor microenvironment [33,34]. The gain of MYB binding to newer sites suggests that its recruitment was likely facilitated through its novel interactions with transcription factors or co-regulators. Indeed, we observed a striking redistribution of MYB binding under hypoxia upon HIF1α deletion. Several MYB peaks that were gained in HIF1α knockout cells under hypoxia overlap with MYB peaks observed in normoxic conditions, suggesting a role of HIF1α in MYB binding at these sites under hypoxic environment. Earlier studies suggest that HIF1α exhibits pioneer-like factor activity, initiating chromatin remodeling and rendering inaccessible DNA regions amenable to other transcription factors by recruiting histone modifiers [35,36]. This ability to ‘open’ chromatin is crucial for expanding the repertoire of hypoxia-responsive genes by creating a more permissive, yet selective, transcriptional environment [37,38]. Context-dependent interactions of HIF1α with other transcription factors result in synergistic or antagonistic effects, ultimately dictating the specificity and magnitude of the cellular response to hypoxic stress [35, 39].

Interestingly, we noticed that the altered genomic occupancy of MYB was not completely dependent on HIF1α. Our *de novo* motif enrichment analysis near MYB binding sequence identified binding regions for other transcription factors such as NFkB-p50 and YY1, that are implicated in hypoxia adaptation independent of HIF1α [40,41]. Under hypoxic stress, NF-κB, a pivotal transcription factor, orchestrates a complex transcriptional response by not only directly influencing gene expression but also by modulating the chromatin accessibility. Notably, it is shown that NF-κB recruits other transcription factors, including HIF-1α, to integrate innate immunity with the hypoxic response [42]. Yin yang 1 (YY1), a transcription factor exhibits HIF1α-independent activity, directly regulating the PGC-1β, and thereby facilitating lipid accumulation (a key hypoxic adaptation) under both hypoxia and normoxia conditions [41].

In our study, we also observed that for a subset of genes, such as *GATA2* and *INSIG1,* MYB continued to bind at their promoter regions under both normoxia and hypoxia. This persistent MYB binding may represent core regulatory elements crucial for pancreatic cancer cell survival and adaptation [43,44]. *GATA2* is a nuclear transcription factor crucial for the proliferation and maintenance of hematopoietic stem cells and other multipotential progenitor cells [45]. Its expression is required for malignant progression and metastatic potential of cancer cells [46]. INSIG1, a transmembrane protein in the endoplasmic reticulum, is central to cholesterol and fatty acid biosynthesis regulation [47]. In hypoxic conditions, it is shown that the upregulation of *INSIG1* triggers a cascade, leading to the increased expression of EMT-associated genes [48]. The hypoxia-induced increased binding of MYB to specific gene promoters, such as *DDIT4*, and *XBP1* and decreased binding to *CCNA1*, *EEF1A2* suggests its involvement in regulating key genes associated with cell cycle, stress response, and survival pathways in hypoxia [49–52].

Another significant finding in our study was that MYB also had a profound impact on HIF1α genomic distribution. This is in alignment with our previous findings where we observed that MYB enhanced the recruitment of HIF1α to glycolytic gene promoters leading to their synergically induced expression [10]. The pioneering factor activity of MYB has been documented in earlier reports, wherein it recruits chromatin-modifying enzymes, such as p300, leading to histone acetylation and increased chromatin accessibility [29,53]. Interestingly, HIF1α exhibited surprising increase in the binding events upon MYB deletion, suggesting a role of MYB in defining genomic specificity of HIF1α, especially in the proximal promoter regions. Indeed, we have previously reported MYB interaction with p300 that facilitated HIFα binding to the glycolytic gene promoters [10]. Therefore, studying a role of p300 in mediating the impact of MYB-HIF1α crosstalk leading to their altered genomic occupancy is crucial to identifying targetable therapeutic vulnerabilities. RNA-seq followed by pathway analysis of control and MYB knockout cells under hypoxia revealed that MYB regulated a specific subset of hypoxia-induced genes involved in axon guidance and metabolic pathways, including fructose and mannose metabolism, inositol phosphate metabolism, glycolysis and gluconeogenesis. Semaphorins, axon guidance proteins are shown to promote cancer cell migration and metastatic potential [54]. Fructose utilization in pancreatic cancer cells under hypoxic conditions promotes survival and inhibits autophagic cell death [55]. Mannose primarily used for N-linked glycosylation of proteins and it has been shown that hypoxia alters glycosylation levels of secreted glycoproteins that could enhance metastatic capabilities [56]. Also, hypoxia induced increased in cytosolic calcium stimulates the inositol phosphate formation and promotes the aggressiveness of cancer cells [57,58].

The significant overlap in MYB and HIF1α binding sites, particularly at promoters, and concomitant overlap of hypoxia-induced genes suggests the importance of their co-occupancy for hypoxia-induced transcriptional regulation. The finding that approximately ~36 % of HIF1α-regulated genes are also regulated by MYB reinforces the idea of coordinated control for genes of specific pathobiological significance. KEGG pathway analysis identified Hedgehog, axon guidance, hippo and Wnt signaling pathways as the ones that are induced through MYB-HIF1α regulatory crosstalk. This is significant since hedgehog signaling is shown to facilitate hypoxia-induced EMT and invasion of pancreatic cancer cells [59]. Under hypoxia, enhanced nuclear accumulation of YAP1, a component of Hippo pathway, also promotes EMT and invasiveness of pancreatic cancer cells [60]. Similarly, Wnt signaling promotes EMT, survival, and stemness of pancreatic cancer cells [61]. MYB- and HIF1α-co-regulated direct target, *KRT19*, exhibits positive correlation with HIF1α under hypoxia [62], while another target, *ALDOA* is shown to support hypoxic survival of liver cancer cells [63]. *RAB27B* (a member of RAS oncogene family) expression is associated with perineural invasion and higher metastatic potential of pancreatic cancer cells [64] and it plays a crucial role in altered exosome shedding and selective loading of exosomal cargo [65]. The higher expression of *GJB5* (Gap Junction β5, a member of the β-type connexin family) is considered a predictor of poor clinical outcomes in pancreatic cancer patients; however, the information about its contribution in pancreatic cancer pathogenesis is scarce [66].

In summary, our findings reveal a pivotal role for MYB in the transcriptional reprogramming of pancreatic cancer cells under hypoxia, supporting their optimal adaptation and aggressive behavior. MYB has a significant impact on the genomic binding of HIF1α, and *vice versa*, with both proteins co-regulating a critical subset of target genes. These genes are highly expressed in pancreatic cancer, showing a positive correlation with disease progression and a negative association with patient survival. These findings are particularly significant given that pancreatic tumors are characteristically hypoxic, a condition that likely contributes to their notoriously high resistance to chemotherapy. Collectively, MYB, its crosstalk with HIF1α, and their coregulated downstream target genes represent attractive targets for therapy and potential biomarkers for risk prediction in pancreatic cancer.

## Supplementary Material

Supplementary Information

Appendix A. Supplementary data

Supplementary data to this article can be found online at https://doi.org/10.1016/j.canlet.2025.217916.

## Data Availability

All transcriptomic datasets supporting the findings of this study, including ChIP-seq (Fastq and Bam files) and RNA-seq (Fastq files) have been deposited in the GEO database under accession number GSE292665 and GSE292666 respectively. Source data are also provided with this paper, and additional supporting data are available upon reasonable request from the lead corresponding author.

## Abbreviations

PC: Pancreatic Cancer
MBS: MYB binding sites
HRE: Hypoxia response elements
FBS: Fetal Bovine Serum
ChIP-seq: Chromatin Immunoprecipitation Sequencing
RNA-seq: RNA sequencing
GSEA: Gene Set Enrichment Analysis
KEEG: Kyoto Encyclopedia of Genes and Genomes
DEGs: Differentially Expressed Genes
IGB: Integrated Genome Browser
TME: Tumor Microenvironment
KRT19: Keratin 19
ALDOA: Aldolase A
CLDN4: Claudin 4
GJB5: Gap Junction Protein Beta 5
RAB27B: Ras-Associated Binding protein 27 B
GEO: Gene Expression Omnibus
