# Supplementary material for: MYB and HIF1α crosstalk drives hypoxia-induced transcriptional reprogramming and adaptive signaling alterations in pancreatic cancer
Source: Cancer Lett. Author manuscript; Available in PMC 2025 Oct 28. (PMC12551948; doi:10.1016/j.canlet.2025.217916)
Supplement: Supplementary Information [file NIHMS2118257-supplement-Supplementary_Information.docx]

**Supplementary Information:**


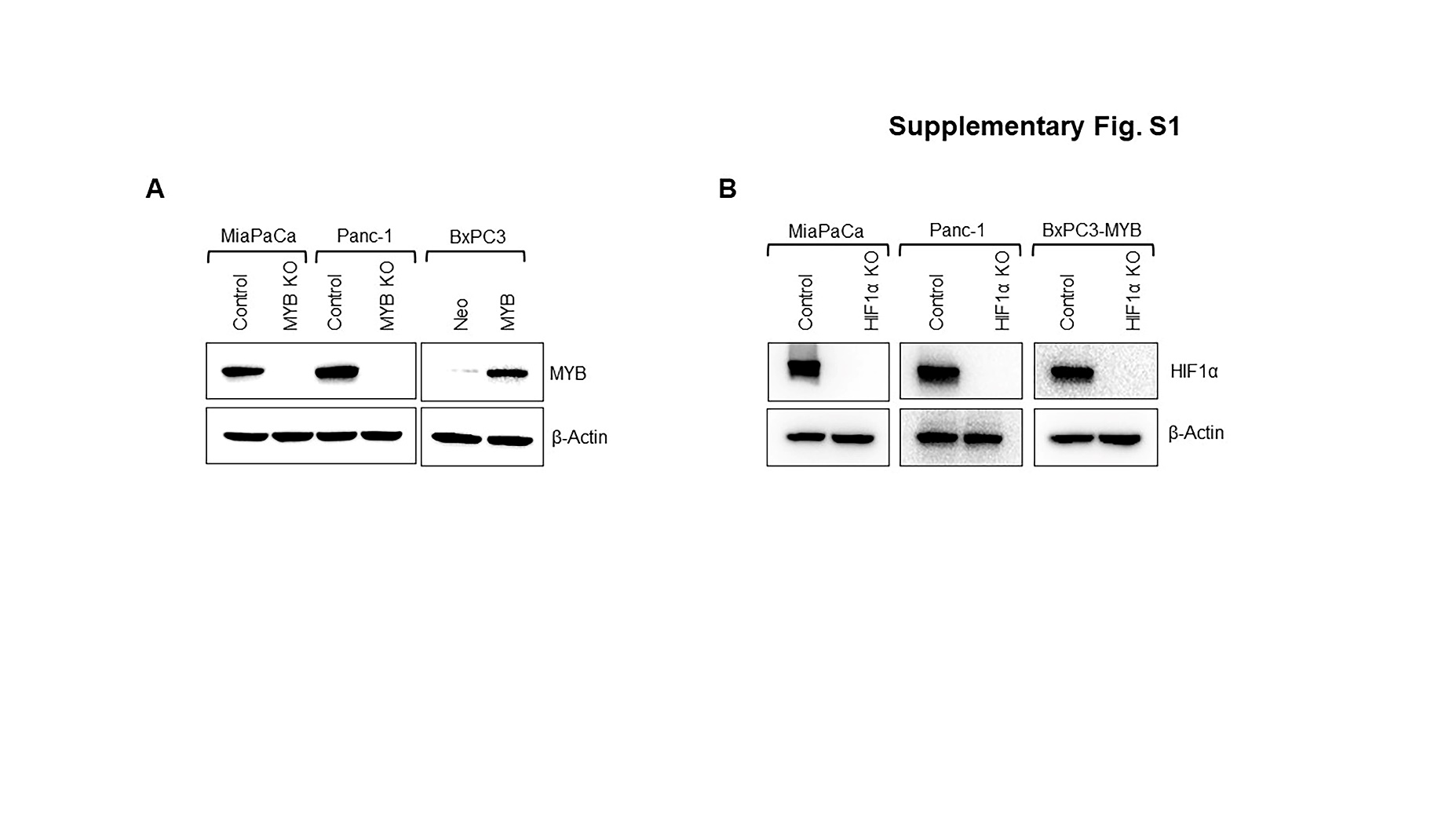


**Supplementary Fig. S1: Validation of MYB and HIF1α expression in genetically modified pancreatic cancer cell lines.** (**A**) Control and MYB knockout MiaPaCa and Panc-1 cells, and forced MYB-expressing cells were generated as described in the Methods section. MYB expression was analyzed by immunoblot analysis. (**B**) HIF1α knockout MiaPaCa, Panc1, and BxPC3 cells were generated and HIF1α expression was examined by immunoblot analysis. β- actin was used as loading control.


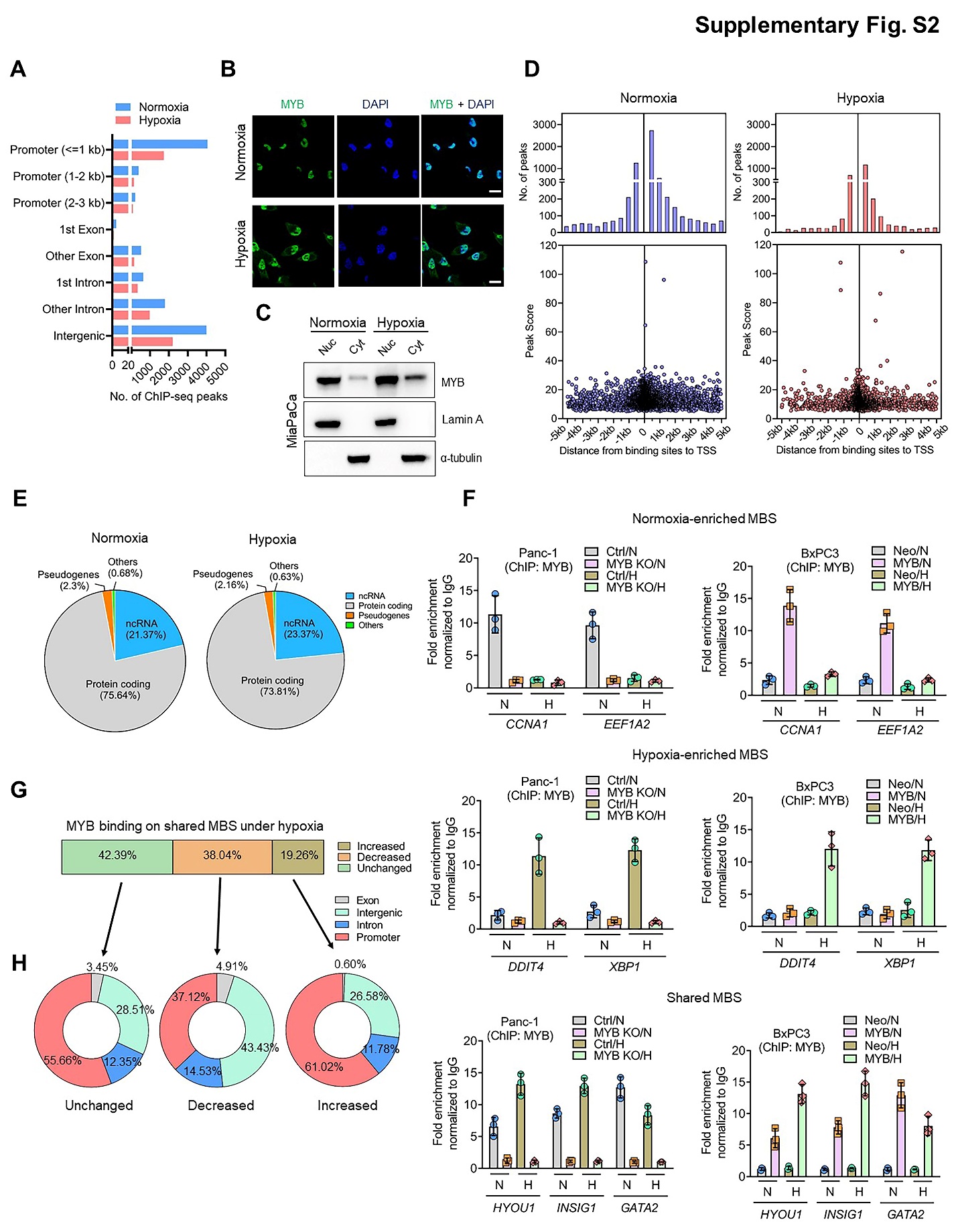


**Supplementary Fig. S2: Analysis of chromatin occupancy of MYB under normoxia and hypoxia.** (**A**) Genomic distribution of MYB ChIP-seq peaks identified from three-independent replicates with significance threshold of -log_10_ (*p-*value > 5) across the defined genomic regions under normoxia and hypoxia (1.0% O_2_, 6h). (**B**) Confocal microscopy analysis of subcellular localization of MYB in cells exposed under normoxia and hypoxia conditions (1.0% O_2_, 6h). MYB (green) nucleus (blue). Scale bar is 25 µm. (**C**) Immunoblot analysis of MYB in nuclear (Nuc) and cytosolic (Cyt) fractions prepared from cells cultured under normoxia and hypoxia for 6h. α-Tubulin and Lamin A were used as a loading control and to assess the purity of subcellular fractions. (**D**) Distribution of number of MYB ChIP-seq peaks (upper panels) and the corresponding peak scores (lower panels) within ±5 kb region of transcription starts site (TSS) at ±1 kb bin intervals were analyzed from MYB ChIP-seq data collected under normoxia (left) and hypoxia (right). (**E**) Biotype distribution profile of all the identified peaks from MYB ChIP-seq analysis under normoxia and hypoxia. Pie chart showing the proportion of protein coding, non-coding RNAs, pseudogenes, and others including rRNAs, snoRNAs, and snRNAs. (**F**) Panc-1 (Ctrl and MYB KO), and BxPC3 (Neo and MYB) cells were exposed under normoxia and hypoxia and subjected to chromatin-immunoprecipitation using either MYB-specific antibody or IgG control followed by qPCR analysis using site‐specific primers. N-Normoxia, H-Hypoxia (**G**) Column graph depicting the percent shared MYB binding sites, where MYB binding was either increased or decreased or remain unchanged under hypoxia compared to normoxia. (**H**) Genomic distribution of shared MYB binding sites categorized into increased (right panel), decreased (middle panel), and unchanged (left panel) occupancy of MYB under hypoxia compared to normoxia.


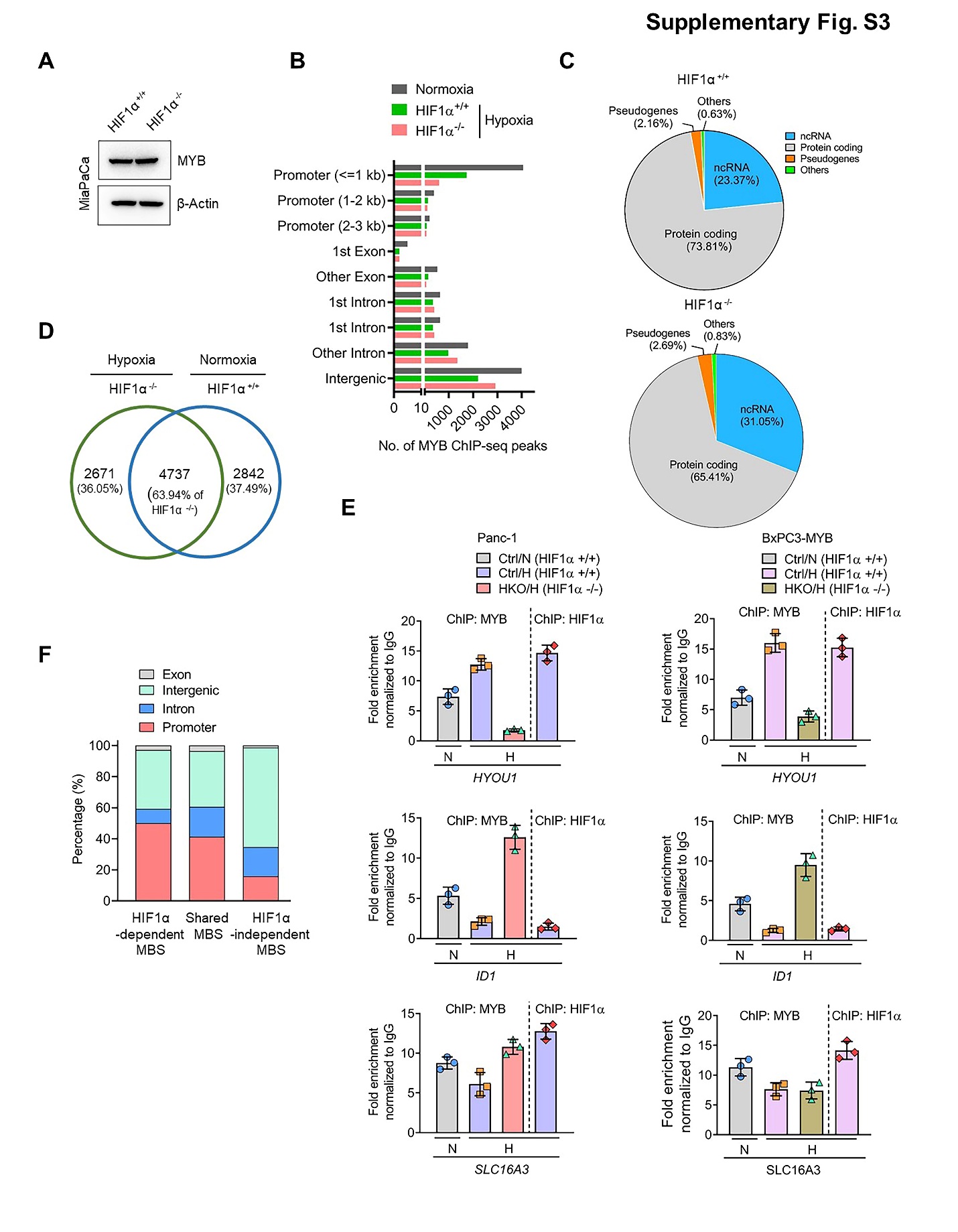


**Supplementary Fig. S3: Role of HIF1α in differential genomic occupancy of MYB under hypoxia.** (**A**) Immunoblot analysis was used to analyze the MYB expression in HIF1α ^+/+^ and HIF1α ^-/-^ cells. β- actin was used as internal loading control. (**B**) Bar diagram depicting the comparison of genomic distribution of MYB peaks (mean of three independent replicates, -log_10_ *p*-value > 5) across the defined genomic regions in HIF1α ^+/+^ cells under normoxia and hypoxia and in HIF1α ^-/-^ cells under hypoxia (1.0% O_2_) only. (**C**) Biotype distribution of MYB peaks identified by MYB ChIP-seq analysis in HIF1α ^+/+^ (upper panel) and HIF1α ^-/-^ cells (lower panel). Pie charts show the percentage proportion of MYB peaks corresponding target genes categorized into protein coding, ncRNAs, pseudogenes, and others including rRNAs, snoRNAs, and snRNAs. (**D**) Venn diagrams showing the overlap between MYB peaks identified in HIF1α ^+/+^ cells under normoxia and HIF1α ^-/-^ cells under hypoxia. (**E**) ChIP-qPCR analysis was conducted to confirm the binding of MYB at promoter region of *HYOU1* and *SLC16A3* and intergenic region of *ID1* in Panc-1 Ctrl and Panc-1 HIF1α KO (HKO) and forced MYB-overexpressing BxPC3 cells (BxPC3-MYB Ctrl) and BxPC3-MYB-HIF1α KO cells. The cells were exposed under hypoxia for 6h and subjected to chromatin-immunoprecipitation followed by qPCR analysis using site‐specific primers. N-Normoxia, H-Hypoxia (**F**) Genomic distribution of MYB binding sites (HIF1α-dependent, shared, and HIF1α-independent) across the defined genomic regions.


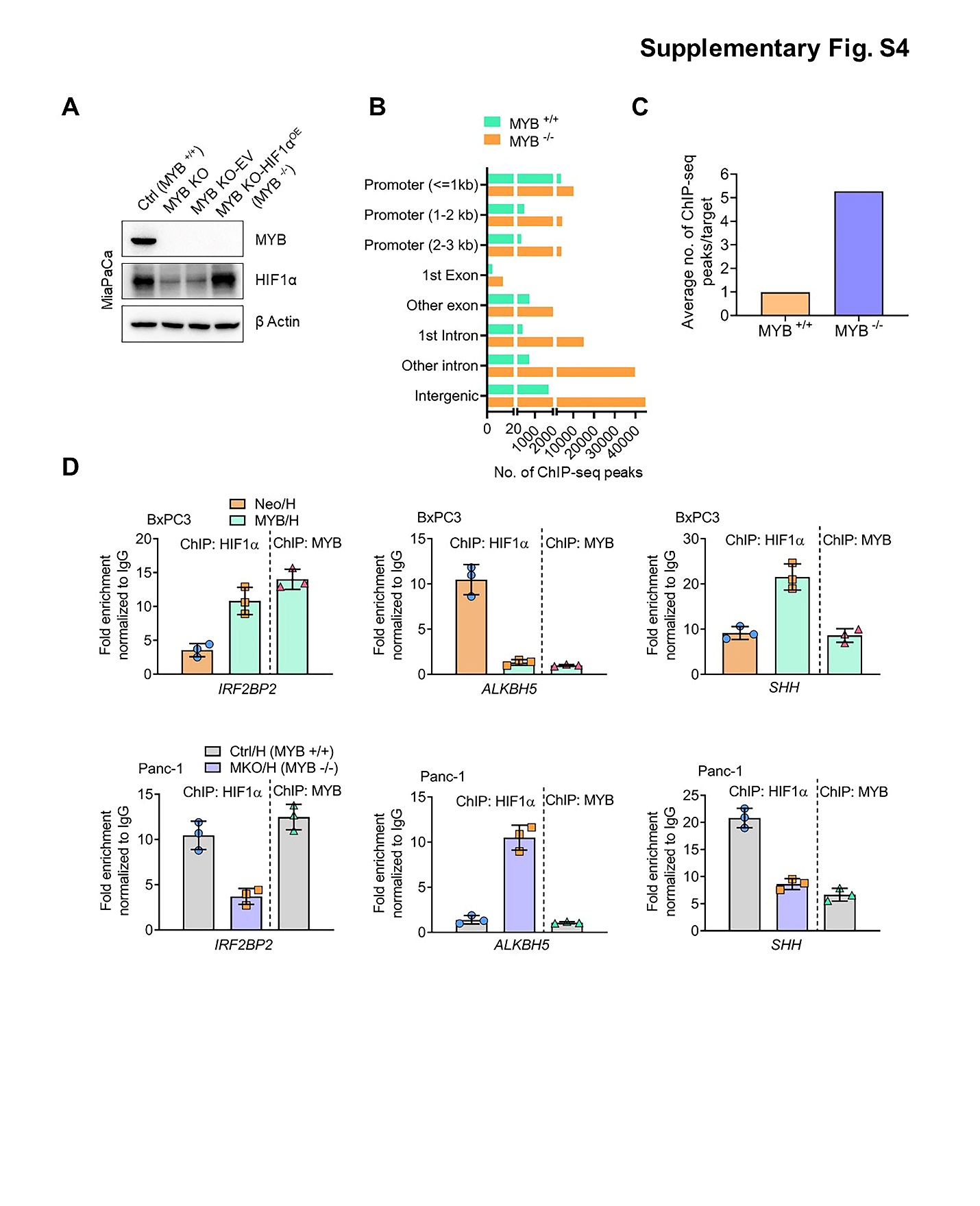


**Supplementary Fig. S4: Effect of MYB deletion on genomic occupancy of HIF1α**. (**A**) Immunoblot analysis of MYB and HIF1α expression in MiaPaCa control (MYB ^+/+^), MYB knockout (MYB KO), MYB knockout cells expressing empty vector (MYB KO-EV), and MYB KO cells with ectopic expression of HIF1α (MYB KO-HIF1α^OE^ or MYB ^-/-^) cultured under hypoxia for 6h. β- actin was used as loading control. (**B**) Genomic distribution of HIF1α binding sites (identified in three independent replicates, -log_10_ *p*-value > 5) across the genome at defined regions in MYB ^+/+^ and MYB ^-/-^ cell cultured under hypoxia (1.0% O_2_) for 6 h. (**C**) Average number of MYB ChIP-seq peaks corresponding to their putative targets were analyzed in MYB ^+/+^ and MYB ^-/-^ cells. (**D**) The binding of HIF1α and MYB at promoter region of *IRF2BP2*, *ALKBH5*, and *SHH* in BxPC3 (Neo and MYB) and Panc-1(MYB ^+/+^ and MYB ^-/-^) cells was confirmed through ChIP-qPCR assay. The cells were exposed under hypoxia (1.0% O_2_) for 6h and subjected to chromatin-immunoprecipitation followed by qPCR analysis using site‐specific primers.


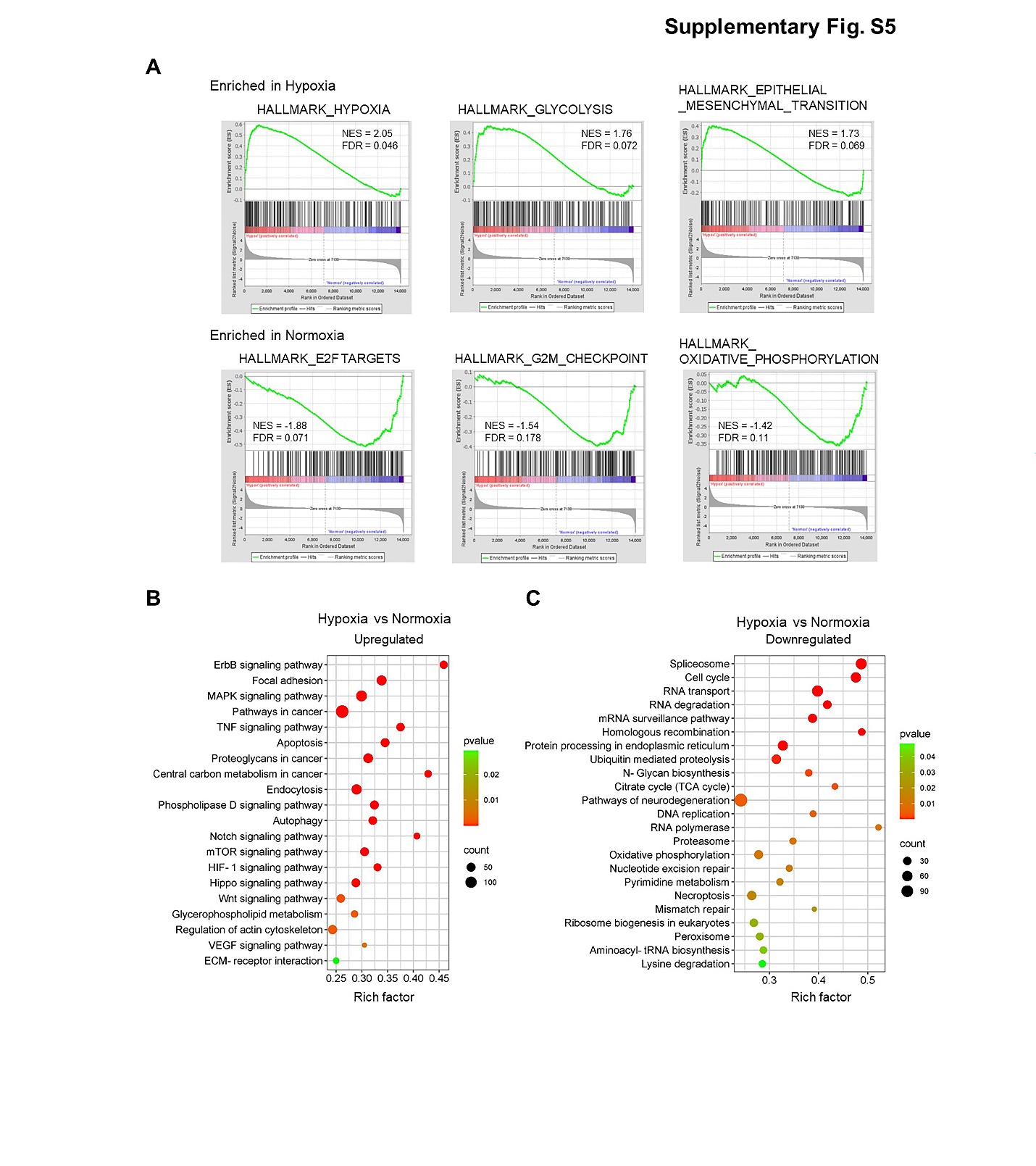


**Supplementary Fig. S5: Pathway enrichment analysis of DEGs under hypoxia**. (**A**) Gene Set Enrichment Analysis (GSEA) was used to determine if hypoxia-induced differentially expressed genes show a statistically significant association with any biological phenotypes. The upper panel shows the significantly enriched pathways associated with genes upregulated under hypoxia and pathways associated with genes showing upregulated expression under normoxia or down regulated under hypoxia are shown in lower panels. Bubble plot of KEGG pathway enrichment analysis of upregulated (**B**) and downregulated (**C**) genes analyzed from RNA-seq analysis (*p-*value < 0.05, FC > 1.5) of pancreatic cancer cells cultured under hypoxia (1.0% O_2_, 24 h).


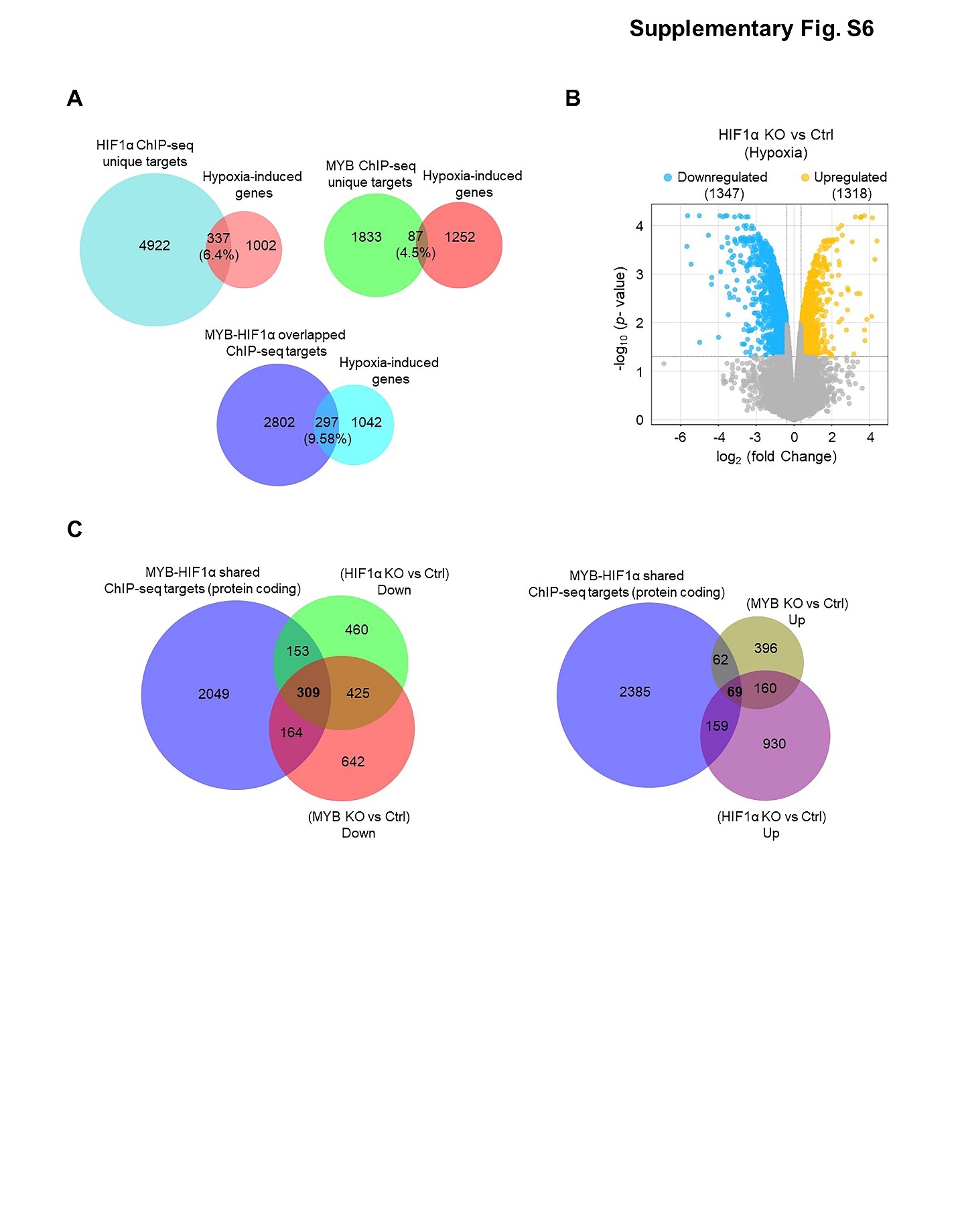


**Supplementary Fig. S6: Comparison of MYB and HIF1α-shared or unique ChIP-seq targets with hypoxia-induced genes or DEGs in MYB KO and HIF1α KO cells.** (**A**) Venn diagram showing the overlap of MYB and HIF1α ChIP-seq unique or shared putative targets with hypoxia-induced genes. (**B**) Volcano plot depicting significant differentially expressed genes identified as a result of bioinformatics analysis of RNAseq data of MiaPaCa ctrl and HIF1α knockout cells. The golden and black dots show significantly upregulated and downregulated genes respectively. The x-axis represents the log_2_ FC and y-axis represents -log_10_ *p*-value. (**C**) Quantitative Venn diagram showing the overlap among MYB-HIF1α shared ChIP-seq targets and downregulated or upregulated genes in HIF1α KO and MYB KO cells.


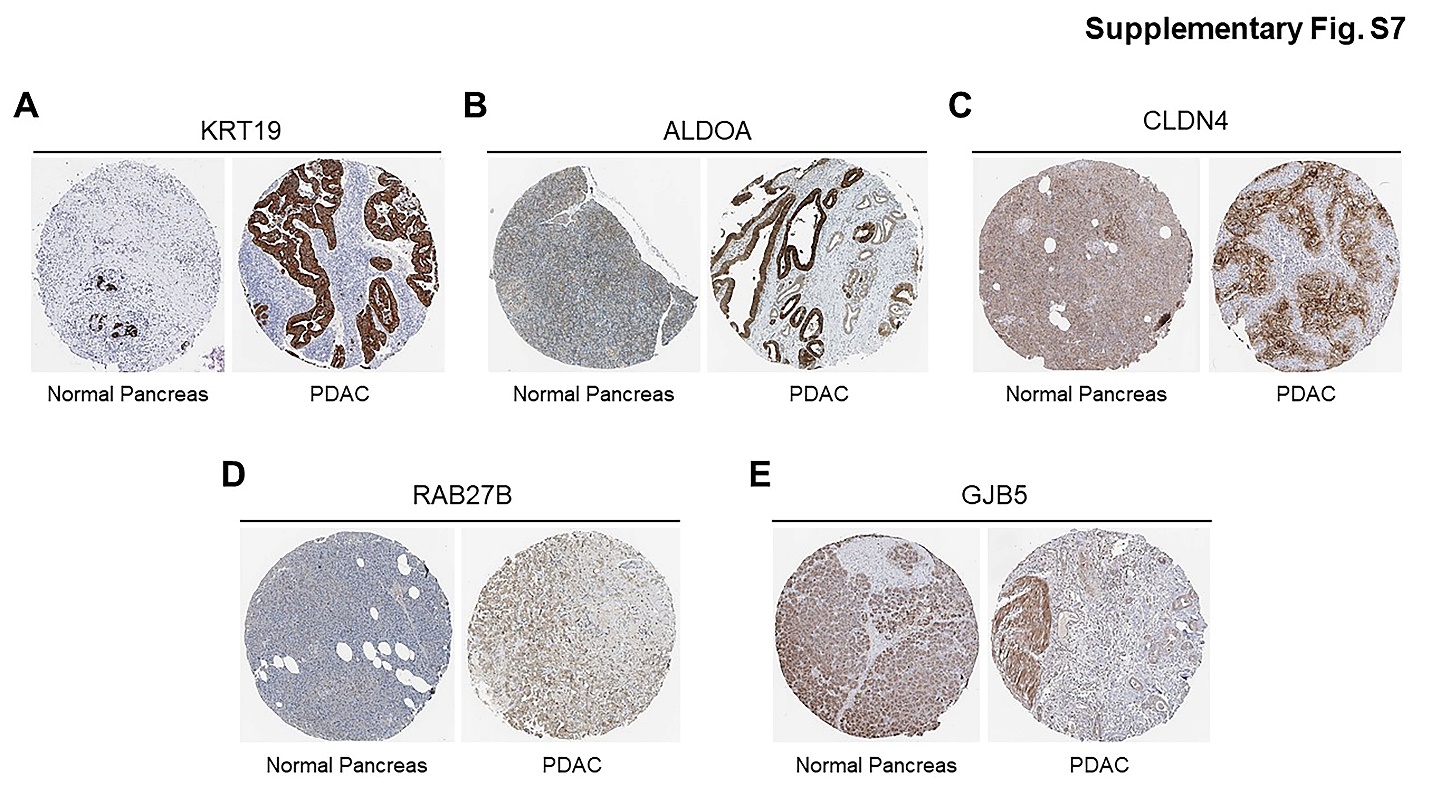


**Supplementary Fig. S7: Immunohistochemical analysis of KRT19, ALDOA, CLDN4, RAB27A, and GJB5 expression in Human Protein Atlas database.** Expression levels of (**A**) *KRT19*, (**B**) *ALDOA*, (**C**) *CLDN4*, (**D**) *RAB27B*, and (**E**) *GJB5* as determined by immunohistochemistry were surveyed in normal pancreas and pancreatic adenocarcinoma tissues in Human Protein Atlas database. Representative images are presented. An overexpression of all the genes is observed in pancreatic cancer with negligible expression reported in the normal pancreas, except for *GJB5*, which also shows a positive staining in the exocrine glandular cells of the normal pancreas.

| **Region or sites** | **Sequence (5ʹ - 3ʹ)** |
| --- | --- |
| CCNA1-F | 5ʹ-GGAGACGCAACACTGCCGCGAC-3ʹ |
| CCNA1-R | 5ʹ-CTGAGAACCCTGGCTCGGGAGAG-3ʹ |
| EEF1A2-F | 5ʹ-GGGCCCGCCGCGCTCTCCCCGC-3ʹ |
| EEF1A2-R | 5ʹ-GGTGGCGGCGCAGTGCACCGCG-3ʹ |
| DDIT4-F | 5ʹ-AAGGCAGCCGGGCGAGGGCCG-3ʹ |
| DDIT4-R | 5ʹ-AAACCCAGCCACGACTGCGGA-3ʹ |
| XBP1-F | 5ʹ-GCTGGGCGGCTGCGGCGCGCG-3ʹ |
| XBP1-R | 5ʹ-CTCCTCGGGGCTCAGGTGCGT-3ʹ |
| HYOU1-F | 5ʹ-GGGCGGGGCCTGGGACGTGGT-3ʹ |
| HYOU1-R | 5ʹ-CCCTTCACAACTCCTCTCGGT-3ʹ |
| INSIG1-F | 5ʹ-CGGTGGCGTGTGCGCACGTCA-3ʹ |
| INSIG1-R | 5ʹ-AGCGCCTGGCGGGCGCGCGGGT-3ʹ |
| GATA2-F | 5ʹ-TGAGCGCCAGGAAGGTAGCGA-3ʹ |
| GATA2-R | 5ʹ-AGGTGCGCGGCAGGCGGGCTC-3ʹ |
| ID1-F | 5ʹ- CCTCCTTGTCACCCAGGCTGG -3ʹ |
| ID1-R | 5ʹ-CACGCCATAGTCCCAGCTACT-3ʹ |
| SLC16A3-F | 5ʹ- GGAGCCCGTCCGGAGGCGGCG -3ʹ |
| SLC16A3-R | 5ʹ-CCCCGGCCCGCGCGCGAGCAT-3ʹ |
| IRF2BP2-F | 5ʹ- GTGGTGCCGGTGGGGGGCGGC -3ʹ |
| IRF2BP2-R | 5ʹ-CGCGCCGTCTCGATGACGAAC-3ʹ |
| ALKBH5-F | 5ʹ- CCAGCCGCTAATTAAGTGACG -3ʹ |
| ALKBH5-R | 5ʹ-CAACAGTGTCGCCGTGCCGCG-3ʹ |
| SHH-F | 5ʹ- CCCTTCTCCTCACCCGCAGAG -3ʹ |
| SHH-R | 5ʹ-GGCGGTGAGCAGCAGGCGCTC-3ʹ |

**Supplementary Table S1**: List of primers for the regulatory region or sites identified after ChIP-seq analyses of MYB and HIF1α.
